# Traditional knowledge and use of wild mushrooms by Mixtecs or *Ñuu savi*, the people of the rain, from Southeastern Mexico

**DOI:** 10.1186/s13002-016-0108-9

**Published:** 2016-09-05

**Authors:** Faustino Hernández Santiago, Jesús Pérez Moreno, Beatriz Xoconostle Cázares, Juan José Almaraz Suárez, Enrique Ojeda Trejo, Gerardo Mata Montes de Oca, Irma Díaz Aguilar

**Affiliations:** 1 Microbiología, Edafología, Campus Montecillo, Colegio de Postgraduados, Km. 36.5 Carretera México-Texcoco, Montecillo, Texcoco, estado de México 56230 Mexico; 2 Departamento de Biotecnología, CINVESTAV, Instituto Politécnico Nacional, Av. Instituto Politécnico Nacional 2508, San Pedro Zacatenco, 07360 México city, Mexico; 3 Instituto de Ecología, A.C., Carretera antigua a Coatepec No. 351, El Haya, 91070 Xalapa, Veracruz Mexico

**Keywords:** Ethnomycology, Edible wild mushrooms, Oaxaca, Biocultural importance, Mycological resources, Oral tradition

## Abstract

**Background:**

Mexico is an important global reservoir of biological and cultural richness and traditional knowledge of wild mushrooms. However, there is a high risk of loss of this knowledge due to the erosion of traditional human cultures which is related with the rapid acculturation linked to high migration of rural populations to cities and the U.S.A., and the loss of natural ecosystems. The Mixtec people, the third largest native group in Mexico only after the Nahua and the Maya, maintain ancient traditions in the use and knowledge of wild mushrooms. Paradoxically, there are few studies of the Mixtec ethnomycology. This study shows our ethnomycological research, mainly focused on knowledge and use of wild mushrooms in communities of the Mixteca Alta, in southeastern Mexico. We hypothesized that among the studied communities those with a combination of higher vegetation cover of natural pine and oak forests, lower soil erosion and higher economic margination had a greater richness and knowledge of wild mushrooms. Our study therefore aimed to record traditional knowledge, use, nomenclature and classification of wild mushrooms in four Mixtec communities and to analyze how these aspects vary according to environmental and cultural conditions among the studied communities.

**Methods:**

In order to analyze the cultural significance of wild mushrooms for the Mixtec people, 116 non-structured and semi-structured interviews were performed from 2009 to 2014. Information about the identified species, particularly the regional nomenclature and classification, their edibility, toxicity and ludic uses, the habitat of useful mushrooms, traditional recipes and criteria to differentiate between toxic and edible species, and mechanisms of knowledge transmission were studied. The research had the important particularity that the first author is Mixtec, native of the study area. A comparative qualitative analysis between the richness of fungal species used locally and the official information of the natural vegetation cover, soil erosion and economic marginalization in each of the studied communities was conducted.

**Results:**

A total of 106 species of mushrooms were identified growing in pine and oak forest, deciduous tropical forest and grassland; among the identified mushrooms we recorded 26 species locally consumed, 18 considered toxic, 6 having ludic uses and the remaining 56 species not being used in the studied areas but some of them having potential as food (56 species) or medicine (28 species). We recorded that 80, 22 and 4 species are ectomycorrhizal, saprotrophic and parasites, respectively. Our study shows that a complex and accurate knowledge related with the use, nomenclature, classification, ecology, gastronomy of wild mushrooms has been developed by Mixtecs; and that there is a relation between natural vegetation cover, lower soil erosion and higher economic marginalization and richness, knowledge and use of mushrooms in the studied communites.

**Conclusion:**

Our study showed that conservation and adaptation of ancestral mycological knowledge survives mainly through oral transmition, maintenance of cultural identity, forest protection, preservation native language and also paradoxically through the current socieconomical marginality among the Mixtec people. We also found that those studied communities with a combination of higher vegetation cover of natural pine and oak forests, lower soil erosion and higher economic marginalization showed a greater richness and knowledge of wild mushrooms. Use and sustainable management of wild mushrooms can be an alternative for local integrated development, but local knowledge and traditional worldview should be included into the regional programs of Mixtec biocultural conservation.

## Spanish abstract

**Introducción:**

México es un importante reservorio mundial de riqueza biológica y cultural y de conocimiento tradicional de los hongos silvestres. Sin embargo, existe un alto riesgo de pérdida de este conocimiento debido a la erosión del conocimiento tradicional de las culturas humanas relacionado con la pérdida de regiones boscosas; rápidos procesos de aculturación; y elevada tasa de migración de la población rural a las ciudades y a los Estados Unidos de América. Los mixtecos, considerado el tercer grupo más numeroso en México, solo después de los nahuas y mayas, mantienen tradiciones ancestrales en el uso y conocimiento de los hongos silvestres. Paradójicamente, existen pocos estudios sobre etnomicología mixteca. En este trabajo se presenta un estudio etnomicológico, principalmente enfocado con el conocimiento y uso de los hongos silvestres en cuatro comunidades mixtecas, ubicadas en la Mixteca Alta, en el sureste de México. Se hipotetizó que las comunidades de estudio con una combinación de mayor cobertura de vegetación natural de bosques de pino y encino, menor grado de erosión del suelo y alto grado de marginación económica poseen un mayor conocimiento tradicional y riqueza de los hongos silvestres. De acuerdo a esto, los objetivos de este estudio fueron registrar el conocimiento tradicional, uso, nomenclatura y clasificación de los hongos silvestres en cuatro comunidades mixtecas y analizar las diferencias de estas variables en relación a aspectos ambientales y culturales entre las comunidades estudiadas.

**Métodos:**

Con el fin de analizar la importancia cultural de los hongos silvestres para los mixtecos, se efectuaron 116 entrevistas no estructuradas y semiestructuradas entre 2009 y 2014. Se registró información relacionada con las especies identificadas, particularmente la nomenclatura y clasificación regional, su comestibilidad, toxicidad y usos lúdicos, el hábitat de los hongos con algún uso, recetas de preparación tradicional y los criterios para diferenciar entre especies tóxicas y comestibles, y los mecanismos de transmisión de conocimiento. La investigación tiene la importante particularidad de que el primer autor es mixteco, nativo de la zona de estudio. Se realizó un análisis cualitativo comparativo entre la riqueza de especies de hongos usados localmente y la información de cifras oficiales de la cubierta de vegetación natural, la erosión del suelo y la marginación económica, para cada una de las comunidades de estudio.

**Resultados:**

Se identificaron 106 especies de hongos silvestres los cuales crecen en los bosques de pino y encino, bosque tropical caducifolio y pastizal; entre las especies de hongos identificados reconocidos por los mixtecos con nombres nativos, 26 se consumen localmente, 18 son consideradas tóxicas, 6 tienen uso lúdico, y las 56 especies restantes no se utilizan en las áreas estudiadas, pero tienen potencial como alimento (56 especies) o medicina (28 especies). Se registraron 80, 22 y 4 especies ectomicorrízicas, saprobias y parásitas, respectivamente. Nuestro estudio muestra que los mixtecos han desarrollado un complejo y preciso conocimiento relacionado con el uso, la nomenclatura, clasificación, ecología y gastronomía de los hongos silvestres en las comunidades estudiadas; y que existe una relación entre la cubierta de la vegetación natural, la erosión del suelo, y la alta marginación económica, con respecto a la riqueza, conocimiento y uso de los hongos silvestres en las comunidades estudiadas.

**Conclusión:**

Nuestro estudio demuestra que la conservación y adaptación de los conocimientos ancestrales micológicos todavía perviven principalmente a través de la transmisión oral, preservación de la identidad cultural, la protección de las áreas boscosas, mantenimiento de la lengua mixteca y también, paradójicamente, a través de la marginalidad socioeconómica en la que actualmente viven los mixtecos. Asimismo, se encontró que aquellas comunidades estudiadas que presentan una combinación de una mayor cobertura de vegetación natural de pino y encino, menor erosión del suelo, y un alto grado de marginación económica mostraron un mayor conocimiento y riqueza de los hongos silvestres.El uso y manejo sostenible de los hongos silvestres pueden ser una alternativa para el desarrollo local integrado, sin embargo el conocimiento y cosmovisión tradicional del mundo deben ser incluidos en los programas regionales de conservación biocultural mixteca.

**Palabras clave:**

Etnomicología, hongos silvestres comestibles, Oaxaca, importancia biocultural, recursos micológicos, tradición oral.

## Background

Mexico is considered a megadiverse country since it harbours approximately 10 % of the terrestrial biodiversity of the planet (1.8 million species) [1]. Regarding the diversity of fungi, Hawksworth [2] estimated the occurrence of approximately 1.5 million species worldwide, while Guzman [3] estimated that there might be over 200,000 species of fungi in Mexico, but only 4 % of the Mexican species have been formally described. Additionally, Mexico is a multicultural country, with more than 60 ethnic groups [4]. Each of these ethnic groups has its own language, worldview and management practices of natural resources. From such biological and cultural diversity, more than 12 ethnic groups inhabiting temperate and tropical areas of Mexico exhibit mycophilic tendencies and deep traditional mycological knowledge [5], including edible, medicinal, ludic (i.e. decorations, handcrafts and toys) and religious-ceremonial uses [6–10]. The Mixtec group, native from sotheastern Mexico, considered to be the indigenous group with the third largest number of speakers in Mexico [4], only after the Nahua and the Maya people. Paradoxically, no detailed ethnomycological studies have been published despite the importance of fungi to this group being documented in both ancient and colonial manuscripts. However, is important to mention that there have been detailed ethnobotanical studies among Mixtec people, showing that they gather more than 90 edible plant species, including vegetables, fruits and roots [11, 12].

The Mixtec people are settled in a vast territory covering the states of Oaxaca, Puebla and Guerrero. The word *Mixtec* comes from the Nahuatl language, meaning “*people of Mixtlan or place of the clouds*”. However, the Mixtec people call themselves *ñuu savi,* meaning “*people of the rain*” [13]. The Mixtec language, which belongs to the Otomanguean languages, is a tonal language in which the meanings of words change depending on their pronunciation tone; and there are 81 Mixtec language variants [4]. The importance of mushrooms for the Mixtec group has been documented in both ancient and colonial manuscripts, including: i) The *Codex Yuta Tnoho,* which probably has its origin in Tilantongo, in the Mixteca Alta of Oaxaca [14]. This is considered the first documentary record linked to the cultural importance of mushrooms in Mexico, includes a history of more than 500 years and describes, in pictographs, the mythical origins of the Mixtec universe and the rituals associated with maize, pulque and sacred mushrooms that led to the first sunrise in the current era [15]; ii) several documents held by the General National Archive *(Archivo General de la Nación*), currently located in Mexico City, dating from 1545 clearly mentioned the persistence of pre-Hispanic religious practices, including the ceremonial use of mushrooms, in the beginning of the colonial period in the Mixtec region of Yanhuitlán, Oaxaca. In an inquisitorial process carried out from 1544 to 1546 against Don Francisco, a local Mixtec governor, the mushroom consumption associated with religious ceremonies are explicitly mentioned and considered and classified as paganism, idolatry and witchcraft by the Spanish Inquisition [16]; iii) The *Canvas of Zacatepec,* painted between 1540 and 1560, includes a glyph with a man with mushrooms on his head [17]. The head rests on the top of a hill, which has been interpreted as a sacred place where ceremonies with mushrooms were held [18].

We report here the results of an ethnomycological study about the indigenous nomenclature and classification; and use of wild mushrooms in four communities of the Mixteca Alta of Oaxaca region, located in the southeastern of Mexico. This type of knowledge is currently generating high interest due to the strong transculturation processes currently happening, and the consequent loss of tradicional knowledge. In addition, because of the nutritional and medicinal properties of fungi, their increasing commercial rating, their enormous ecological importance and the great biotechnological potential of mycological resources. Finally, we also evaluated the hypothesis that Mixtec communities in the Mixteca Alta of Oaxaca in southeastern Mexico, with a combination of higher natural vegetation cover of natural pine and oak forests, lower soil erosion and higher economic margination had a greater richness and knowledge of wild mushrooms compared with those communities lacking this combination of ecological and cultural characteristics.

## Methods

### Study area

**Fig. 1:**
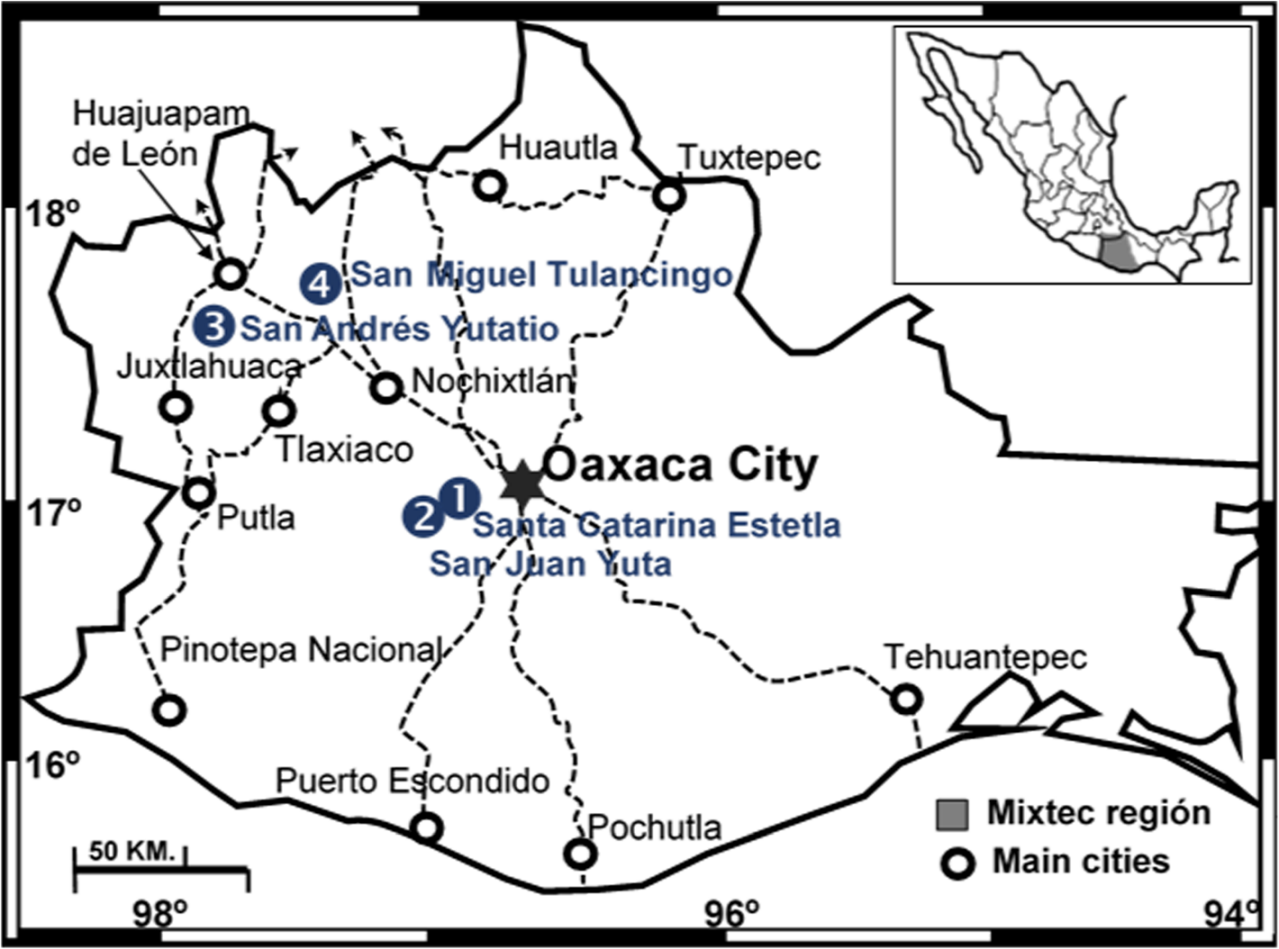
Location of the studied localities marked with numbers from 1 to 4

The study communities are located in the Mixteca Alta region of Oaxaca, Mexico (Fig. 1). The San Juan Yuta community is located south of the municipality of San Juan Tamazola, Oaxaca, located at the geographical coordinates 17° 01′ 23″ N and 97° 10′ 05″ W at 1640 m a.s.l. The predominant climate is (A)C(w) (semi-warm sub-humid), with temperatures of 16–22 °C and summer rains. The Santa Catarina Estetla community is located southwest of the Santa María Peñoles municipality, Oaxaca, at 17° 01′ 35.63″ N and 97° 05′ 50.33″ W, at 2000 m a.s.l. The predominant climate is (A)C(w) (semi-warm sub-humid), with temperatures of 14–22 °C and summer rains. The community of San Andrés Yutatío is located in the southwestern portion of the municipality of Teozatlán de Segura y Luna, Oaxaca, at 17° 36′ 29.05″ N and 97° 53′ 37.91″ W, at 2000 m a.s.l. The predominant climate is C(w) (temperate sub-humid), with temperatures from 16 to 24 °C and summer rains. The community of San Miguel Tulancingo is located at the northwest of the city of Oaxaca at 17° 45′ 1.77″ N and 97° 26′ 29.40″ W, at an altitude between 2000 and 2700 m a.s.l. The predominant climate is C(w) (temperate sub-humid), with temperatures from 14 to 18 °C and summer rains. All of the climates categories of the studied communities were based on Köppen’s classification system [19].

The outstanding vegetation types in the communities of the study, following the classification of Rzedowski [20], are *Pinus* and *Quercus* forests, xerophytic scrubs (primarily sclerophyllous), palm groves and small areas of tropical deciduous forest. In the *Pinus* and *Quercus* forests, 12 species of pines and at least 15 oaks are recorded, with *Pinus oaxacana* and *P. lawsonii* among the most abundant trees. Secondary grasslands are located in small areas of these communities, composed by grasses, sedges and small annual herbs. The evergreen sclerophyllous shrubs are floristically very rich. The species that occur most frequently are *Comarostaphylis polifolia* and *Forestiera rotundifolia*. Secondary palm groves of *Brahea dulcis* and *Brahea nitida* occur in areas that are subject to periodic burning and logging of the oak forest. The tropical deciduous forest is dominated by species of *Bursera* and *Pachycereus*. The agriculture of Mixtec communities is of the subsistence and marginal types. Corn (*Zea mays* L.), beans (*Phaseolus vulgaris* L.), pumpkin (*Cucurbita* spp.*)* and chili peppers (*Capsicun annuum* L.) are the basis of their diet. The diet of the Mixtec peoples in these regions is also complemented by animal husbandry and collected food, primarily more than 90 edible plant species, including vegetables, fruits and roots [11, 12], wild mushrooms as well as, to a lesser extent, hunting and insect gathering.

### Ethnomycological work

During the rainy seasons from 2009 to 2014, field observations were conducted with the company of persons who were recognized by the community as having greater knowledge of wild forest elements, particularly fungi. These persons were selected through the “snowball” technique following a theoretical sampling methodology following Sandoval [21]. Different vegetation types were toured, and wild mushrooms of cultural importance were collected. Additionally photographs and fresh collected specimens were shown to participants to collect information. Under a method of participant observation, informal unstructured and semi-structured interviews were conducted with the informants [22]. Four annual visits were conducted in each community during the rainy season from February to September, during which 116 semi-structured and non-structured interviews were performed. In these interviews the following information was obtained : i) socio-demographic information including gender, age, language and community of residence of interviewees; ii) recognized fungal species; ii) local nomenclature and classification; iii) edible species; iv) species considered toxic; v) species with ludic use; vi) habitat of the fungi locally used; vi) mechanisms of transmission of knowledge; vi) traditional forms of mushroom preparation; vii) criteria of differentiation between edible and poisonous species. To test the hypothesis, official information published by Mexican Government Agencies presenting regional and local information of the studied communities related with the natural vegetation cover, soil erosion and economic marginalization were used [19, 23, 24]. This information was compared with the information gathered in the present work related to the richness of fungal species locally used in each of the four studied communities, which was indicative of the local traditional knowledge and use.

The collected specimens were photographed, their macro- and micromorphological features were described and the specimens dried for preservation [25]. Taxonomic identifications of the material were performed using the techniques proposed by Largent et al. [26] and Tulloss [27] along with the consultation of other studies [28–35], among others. The nomenclature of scientific names of fungi was based on the Index Fungorum [36] and on the plants in the USDA database [37]. The labelled specimens were deposited in the Mycological Collection of the Department of Microbiology of the Postgraduate College, in Montecillo, State of Mexico.

In addition, a review of literature was conducted to collect traditional names for mushrooms in different linguistic variants of Mixtec in Oaxaca. For this activity, vocabularies and Mixtec-Spanish dictionaries published by the Summer Institute of Linguistics in Mexico were the primary materials reviewed [38–48]. The etymological interpretation of the information presented in these dictionaries was always made in the context of the studies that cited the words related to mushrooms, other studies published in the area [as the Mixtec language has 81 language variations [4] and the meanings of words change between these variants] or based on ethnomycological research conducted in the region from 2009 to 2014, which was facilitated by the fact that the first author is a Mixtec speaker and native of the study area. The writing of the different terms in the Mixtec language was based on the nomenclature proposed by some Mixtec-Spanish dictionaries [41–45].

## Results

The 116 people to which unstructured and semi-structured interviews were conducted to people of the following age ranges: 34 % were under 20 years old, 34 % between 20 and 40 years old; 12 % between 40 and 60 years old and 20 % over 60 years old. Fifty six percent of interviewees were men and 44 % were women; 98 % were bilingual Spanish and Mixtec speakers and 2 % were only Mixtec speakers. Fifty one percent, 34, 8 and 7 % of the interviewees inhabit the localities of Santa Catarina Estetla, San Juan Yuta, San Miguel Tulancingo and San Andrés Yutatío, respectively.

**Fig. 2:**
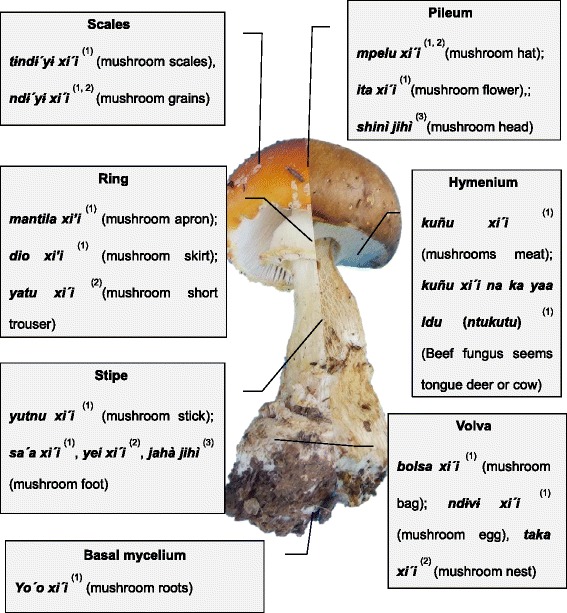
Structures of Agaricales and Boletales mushrooms distinguished by the people of Santa Catarina Estetla (1), San Juan Yuta (2) and Chalcatongo (3) in the Mixteca Alta, Oaxaca, Mexico

In general, the names designating species of mushrooms in the Mixtec language are made up of two words, a root that usually means “mushroom” and a modifier that can be an adjective or noun. These modifiers generally indicate a quality of the mushroom or its similarity to some element of the environment. Sometimes the modifiers indicate an ecological relationship of a particular species. In the case of macrofungi, or wild mushrooms, the generic name is "***xi'i***". This word can be literally translated as "dead or dying", perhaps related to the relatively short longevity of sporocarps. Traditional knowledge of mushrooms among the studied communities is of high accuracy from the western taxonomic and ecological perspective. People can distinguish and name the parts of these species in the local language (Fig. 2); group them and assign one or two names in Mixtec to the most common, edible or poisonous mushrooms; and pinpoint exactly the habitat and phenology of the species studied. Furthermore, mushrooms are separated as organisms distinct from plants and animals (Table 1). The different types of mushrooms are named with suffixes that refer to a particular feature, being associated with a familiar concept in their known universe (flowers, animals, colours, smells, habitats, etc.).

**Table 1:** Classification of living things by the Mixtecs of Santa Catarina Estetla, Oaxaca

General classification	Mixtec name	Examples of subclasses and Mixtec name
Animals	*kɨtɨ, tɨ-*	Animal house (domestic) “*kɨtɨ tata*”; animal field (wild) “*kɨtɨ yuku*”; animal harmful (injurious) “*kɨtɨ kui´na*”
Trees and shrubs^a^	*yutnu, tnu-*	Pine tree “*tnuyusa*”, oak “*tnuyaa*”, chamizo “*tnutau*”, carrizo “*tnuyoo*”
Herbs	*yɨ´ɨ, ku´u, yukú*	Herbs “*ku´u*”, flower “*ita*”, grass “*ite*”
Mushrooms	*xi´i*	Good mushroom (edible mushroom) “*xi´i va´a*”, mushroom that is eaten *(*“*xi´i saxio*”*)*
		Mushroom bad (poisonous mushroon) or crazy mushroom “*xi´i kue´e*”, mushroom that is not eaten “*xi´i un tu saxío*” Frog mushroom “*xi´i la´va*”, Toad mushroom “*xi´i la´va ndɨ´yɨ*”
		Mushroom growing on the dry stump “*xi´i kene nuu ntu´u”,* “*xi´i kene nuu yutnu*”
		Mushroom that grows on the soil “*xi´i kene nuu ñu´u*”
		Mushroom that grows on manure “*xi´i nuu ka´ava*”
		Mushroom that grows on the leaf litter “*xi´i kene nuu vixi*”

^a^Includes shrubs, woody monocots and robust herbaceous plants

A peculiar case in the Mixtec classification system is constituted by a corn parasitic fungus. In this case, the Mixtec name used for the species *Ustilago maydis*, which is a fungus belonging to the Ustilaginales order that is consumed in the region, the word ***xi'i*** is not included because in the studied communities it is not considered to be a mushroom. Instead, the name that refers to the species is ***tɨkaa maa***, which can be translated as "bad grasshopper" and relates to the black colour of the grasshopper (*Sphenarium purpurascens*) or to the dark brown liquid ejected by the mouthparts of these insects, which is similar to the colour of the spores of the fungus.

### Uses of fungi

The most widespread knowledge among the inhabitants of the communities is associated with the use of fungi as food; however, there is also knowledge associated with toxic fungi and with the ludic use of some species.

#### Edible mushrooms and preparation methods

**Fig. 3:**
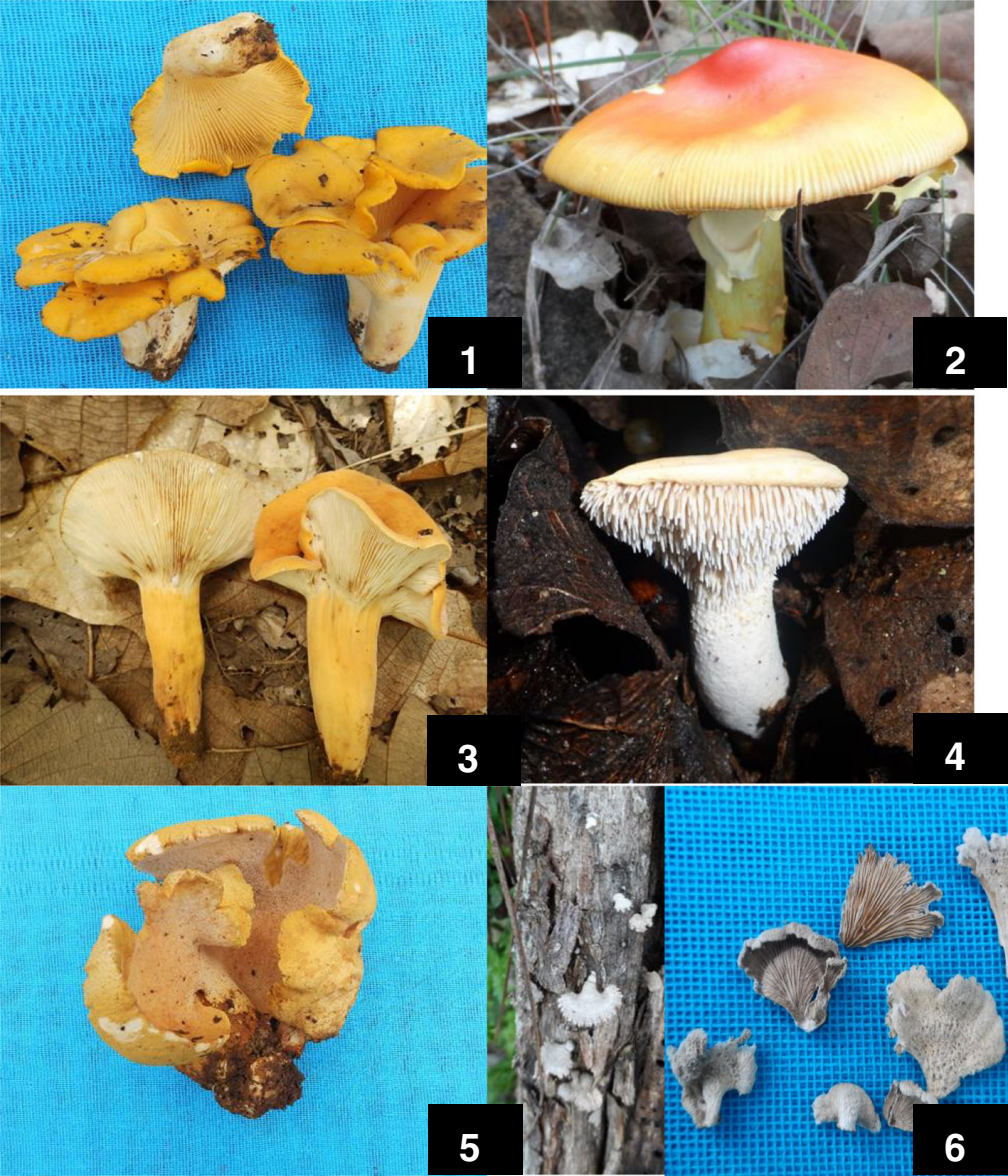
Edible mushrooms in Santa Catarina Estetla and San Juan Yuta, state of Oaxaca, Mexico. **1**. *Cantharellus cibarius s. l.*; **2**. *Amanita* aff*. jacksonii*; **3**. *Lactarius volemus*; **4**. *Hydnum repandum*; **5**. *Albatrellus* aff. *ovinus*; and **6**. *Schizophyllum commune*

**Fig. 4:**
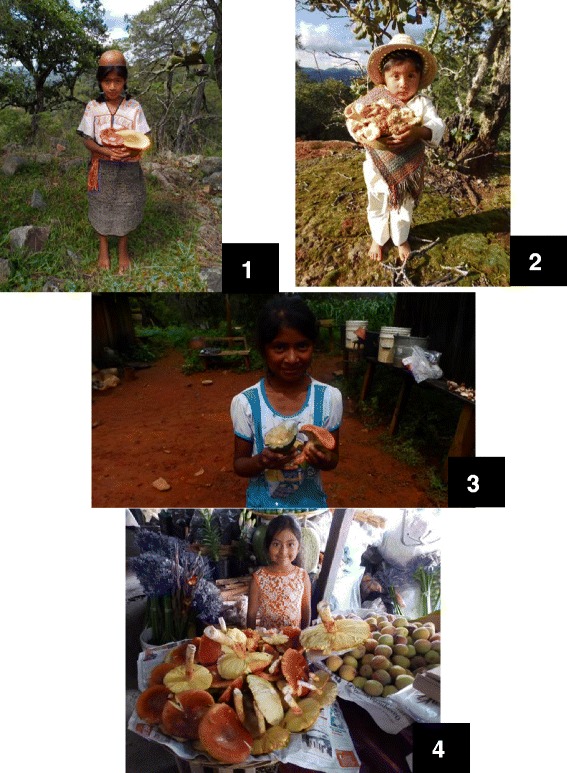
Gathering of wild edible mushrooms by Mixtec children in “Mixteca Alta Oriental” of Oaxaca (**1**, **2**, **3**); collecting wild edible mushrooms by the population of the communities studied (**4**); marketing of *A.* aff. *jacksonii* in the market of the city of Oaxaca, Mexico (**5**)

Of the available wild mushrooms in the study area, respondents said to consume 26 species (Tables 2 and 3, Figs. 3 and 4). Within the complex *Amanita sect. caesarea* (***xi´i naa***), the inhabitants of Santa Catarina Estetla and San Juan Yuta usually identify sporocarps through morphological characters such as their yellow-orange and red colour which include species such as *A.* aff. *basii*, *A*. aff. *jacksonii* and *A*. aff. *laurae*. Commonly, the species of this complex are referenced to as a single taxon. The taxon is characterized by the red, orange or yellow colour of its pileus, yellow lamellae, a yellow ring on the stem, a characteristic odour and because they are born from an ***ndɨvɨ*** or "egg". However, people collected them with caution because some specimens of *Amanita muscaria* (***xi´i la´va ndɨ´yɨ***) may be confused because of their age or because rain can wash away the colour and scales from the pileus.

**Table 2:** Species of wild edible mushrooms in the study communities

Taxa	Mixtec name	English translation
*Agaricus campestris* L. ex Fr.	*xi´i nuu ite* (*xi´i* = mushroom; *nuu* = above; *ite* = grass)	grass mushroom
*Agaricus pampeanus* Speg.	*xi´i ndeyu (ndei)* (*xi´i* = mushroom; *ndeyu* = mole “amarillito”)	mushroom use to prepare mole “amarillito”
*Albatrellus* aff. *ovinus* Schaeff.	*xi´i yaa idu* (xi´i = mushroom; *yaa* = tongue; *idu* = deer (*Odocoileus virginianus oaxacensis*)	deer tongue mushroom
*Amanita* aff. *basii* Guzmán & Ram. Guill.; *Amanita* aff. *jacksonii* Pomerl.; *Amanita* aff. *laurae* Guzmán & Ram. Guill.; *Amanita* sect. *caesarea* (Scop.:Fr.) Pers.	*xi´i naa* (*xi´i* = mushroom; *naa* = exterminate)	mushroom who dies fast
*Boletus edulis s.l.* Bull. ex Fr.	*xi´i taka ya´a* (*xi´i* = mushroom; *taka* = nest of bird; *ya´a* = brown)	brown nest mushroom
*Cantharellus cibarius s.l.* Fr.	*xi ´i veya* (*xi´i* = mushroom; *tɨveya =* pumpkin flower (*Cucurbita* spp.)	pumpkin flower mushroom
*Hohenbuehelia petaloides* (Bull.) Schulzer	*xi´i tnu tɨ´ma* (*xi´i* = mushroom; *tnu =* tree; *tɨ´ma* = cazahuate (*Ipomoea murocoides* Roem. & Schult.)	mushroom of the cazahuate tree
*Hydnum repandum* L.: Fr.	*xi´i tɨntaku* (*xi´i* = mushroom; *tɨndaku =* worm)	worm mushroom
*Hypomyces lactifluorum* (Schw. Fr.)	*xi´i lo´o* (*xi´i* = mushroom; *lo´o =* rooster)	mushroom of rooster
*Calvathia cyathiformis* (Bosc) Morgan	*xi´i ndɨvɨ kuni (xi´i = mushroom; ndɨvɨ = egg; kuni* = turkey hen)	egg mushroom of turkey hen
*Lactarius volemus* Fr*.*	*xi´i dɨkuɨ*(*xi´i* = mushroom; *dɨkuɨ =* milk)	milk mushroom
*Marasmius oreades* Bolt. ex Fr.	*xi´i daa* (*xi´i* = muhroom; tɨ*daa =* bird); *xi´i ndeyu* (*xi´i* = mushroom; *ndeyu =* “amarillito” mole); *xi´i nuu ite* (*xi´i* = mushroom; *nuu* = above; *ite =* grass)	bird mushroom; mushroom used to prepare mole “amarillito”; grass mushroom
*Neolentinus lepideus* (Buxb.) Fr.	*xi´i ntaka’an ñu´u* (*xi´i* = mushroom; *ntaka’an = returns to talk*; *ñu´u* = God or land); *xi´i kolo* (*xi´i* = mushroom; *kolo =* turkey)	mushroom of thunder (when God or land returns to talk); turkey mushroom
*Pleurotus* aff. *eryngii* (Fr.)	*xi´i tnu tɨ´ma* (*xi´i* = mushroom; *tnu (yutnu) =* tree; *tɨ´ma* = cazahuate)	mushroom of cazahuate tree
*Pleurotus* aff. *dryinus* (Pers. ex Fr.) Kum.	*xi´i tnu tɨ´ma* (*xi´i* = mushroom; *tnu (yutnu) =* tree; *tɨ´ma* = cazahuate)	mushroom of cazahuate tree
*Pseudofistulina radicata* (Schw.) Burds.	*xi´i tuchi* (*xi´i* = mushroom; *tuchi =* tendon, leathery) *xi´i tnu xikunta* (*xi´i* = mushroom; *tnu* (*yutnu*) *=* tree; *xikunta* = guachépil (*Diphysa robinioides* Benth.)	mushroom of tendon mushroom of guachépil tree
*Ramaria botrytis (*Pers.) Ricken	*xi´i ndɨkɨ idú* (*xi´i* = mushroom; *ndɨkɨ =* antler; *idú* = deer)	mushroom of antler deer
*Ramaria flava* Quel.	*xi´i ndɨkɨ idú* (*xi´i* = mushroom; *ndɨkɨ =* antler; *idú* = deer)	mushroom of antler deer
*Russula mexicana* Burl.	*xi´i satu* (*xi´i* = mushroom; *satu =* spicy, hot) *xi´i ya’a* (*xi´i* = mushroom; *ya´a =* peper)	spicy mushroom mushroom of pepper
*Schizophyllum commune* (Fr.) Fr.	*xi´i tnu kutu* (*xi´i* = mushroom; *tnu* (*yutnu*) *=* tree; k*utu* = copal (*Bursera* spp.)	mushroom of copal tree
*Ustilago maydis*	*tɨká maa* (*tɨka* = grasshopper; *maa =* bad)	bad grasshopper

**Table 3:** Species of mushrooms used as food, with ludic use or toxic recognized by Mixtecs in the studied communities

Taxa	Mixtec name	Use	Substrate	Habitat	TG	Mixtec community
Ascomycetes						
*Hypomyces lactifluorum* (Schw. Fr.)	*xi´i lo´o*	EL	M	Q, P, P-Q	MY	1, 2
Basidiomycetes						
*Agaricus campestris* L. ex Fr.	*xi´i nu ite*	EL	H	G	S	1, 2
*Agaricus pampeanus* Speg.	*xi´i nde´i*	EL	H	G	S	4
*Albatrellus* aff. *ovinus* Schaeff.	*xi´i yaa idu*	EL	H	P	S	1
*Amanita* aff. *basii* Guzmán & Ram. Guill.	*xi´i naa*	EL	S	Q, P-Q	EM	1, 2
*Amanita bisporigera* G.F. Atk	*xi´i la´ava*	DT	S	Q, P-Q	EM	1, 2
*Amanita chlorinosma* (Peck) Lloyd	*xi´i la´ava ndɨ´yɨ kuixi*	T	S	Q, P, P-Q	EM	1
*Amanita citrina* Pers.	*xi´i la´ava ndɨ´yɨ*	T	S	Q, P-Q	EM	1
*Amanita crocea* (Quél.) Singer ex Singer.	*xi´i la´ava*	T	S	Q, P-Q	EM	1
*Amanita echinocephala* (Vittad.) Quel.	*xi´i la´ava ndɨ´yɨ*	T	S	Q, P-Q	EM	1
*Amanita flavoconia* G.F. Atk.	*xi´i la´ava ndɨ´yɨ*	T	S	Q, P-Q	EM	1
*Amanita flavorubens* (Berk. & Mont.) Sacc.	*xi´i la´ava ndɨ´yɨ*	T	S	Q, P-Q	EM	1
*Amanita fulva* Fr.	*xi´i la´ava*	T	S	Q, P-Q	EM	1
*Amanita gemmata* (Fr.) Bertill.	*xi´i la´ava ndɨ´yɨ*	T	S	Q, P, P-Q	EM	1
*Amanita* aff. *jacksonii* Pomerl.	*xi´i naa*	EL	S	Q, P-Q	EM	1, 2
*Amanita aff. laurae* Guzmán & Ram. Guill.	*xi´i naa*	EL	S	Q, P-Q	EM	1, 2
*Amanita muscaria* (L.: Fr.) Lam.	*xi´i la´ava ndɨ´yɨ*	T	S	Q, P, P-Q	EM	1, 2
*Amanita phalloides* (Vaill. ex Fr.) Link.	*xi´i la´ava*	DT	S	Q, P-Q	EM	1
*Amanita polypyramis* (Berk. & M.A. Curtis) Sacc.	*xi´i la´ava kuixi*	T	S	Q, P-Q	EM	1
*Amanita rubescens* (Pers.: Fr.) S.F. Gray.	*xi´i la´ava ndɨ´yɨ*	T	S	Q, P-Q	EM	1
*Amanita* sect. *caesarea* (Scop.:Fr.) Pers.	*xi´i naa*	EL	S	Q, P-Q	EM	1, 2
*Amanita vaginata* (Bull.) Lam.	*xi´i la´ava*	T	S	Q, P-Q	EM	1
*Amanita verna* (Bull.: Fr.) Lamarck.	*xi´i la´ava*	T	S	Q, P-Q	EM	1
*Amanita virosa* (Fr.) Bertill.	*xi´i la´ava*	DT	S	Q, P, P-Q	EM	1
*Astraeus hygrometricus* (Pers.: Pers.) Morgan.	*xi’i chɨndɨɨ*	L	S	Q, G	EM	1
*Boletus edulis s.l.* Bull. ex Fr.	*xi´i taka ya´a*	EL	S	Q, P-Q	EM	1
*Calvatia* aff. *cyathiformis* (Bosc) Morgan	*xi´i ndɨvɨ kuni*	EL, L	H	G	S	1,3
*Cantharellus “cibarius”* sp. 1	*xi´i veya*	EL	S	Q, P, P-Q	EM	1, 2
*Cantharellus “cibarius”* sp. 2	*xi´i veya*	EL	S	Q, P, P-Q	EM	1, 2
*Hohenbuehelia petaloides* (Bull.) Schulzer.	*xi´i tnu tɨ´ma*	EL	W	TDC	S	1
*Hydnum repandum* L.: Fr.	*xi´i tɨntaku*	EL	S	Q, P-Q	EM	1
*Lactarius volemus* Fr.	*xi´i dɨkuɨ*	EL	S	Q, P-Q	EM	1, 2
*Lepiota sp.*	*xi´i la´ava ndɨ´yɨ*	T	H	Q, P-Q	S	1
*Lycoperdon pyriforme* Schaeff.	*xi’i kue´e*	L	H	G	S	1, 2
*Lycoperdon* aff. *spadiceum* Pers.	*xi’i kue´e*	L	H	G	S	1
*Lycoperdon perlatum* Pers.	*xi’i kue´e*	L	H	G	S	1, 2
*Macrolepiota procera* (Scop.) Singer	*xi´i la´ava ndɨ´yɨ*	T	H	P	S	1, 2
*Marasmius oreades* Bolt. ex Fr.	*xi´i daa, xi´i ndeyu*	EL	H	G	S	1, 2
*Neolentinus lepideus* (Buxb.) Fr.	*xi´i ntaka’a ñu´u; xi´i kolo*	EL	W	P, P-Q	S	1, 2
*Pisolithus arhizus* (Scop.: Pers.) Rausch.	*xi’i ndɨvɨ burru*	L	S	P, P-Q	EM	1
*Pleurotus* aff. *dryinus* (Pers. ex Fr.) Kum.	*xi´i tnu tɨ´ma*	EL	W	TDF	S	1, 2
*Pleurotus* aff. *eryngii* (Fr.)	*xi´i tnu tɨ´ma*	EL	W	TDF	S	1, 2
*Pseudofistulina radicata* (Schw.) Burds.	*xi´i tuchi*	EL	W	Q, P-Q	S	1, 2
*Ramaria* aff. *fennica* (P. Karst.) Ricken	*xi´i ndɨkɨ idu*	EL	S	Q	EM	1, 2
*Ramaria botrytis* (Pers.) Ricken.	*xi´i ndɨkɨ idu*	EL	S	Q	EM	1, 2
*Ramaria flava* Quel.	*xi´i ndɨkɨ idu*	EL	S	Q	EM	1, 2
*Russula mexicana* Burl.	*xi´i satu, xi´i ya’a*	EL	S	Q, P-Q	EM	1
*Schizophyllum commune* (Fr.) Fr.	*xi´i tnu kutu*	EL	W	TDF	S	1, 2
*Ustilago maydis* (DC.) Corda.	*tikaa maa*	EL	O	C	P	1, 2, 3, 4

The nomenclature is based on *the Index fungorum* [36] and the ectomycorrhizal status in Rinaldi et al. [96] and Comandini et al. [97]. *EL* edible locally, *L* ludic, *T* toxic, *DT* deadly toxic, *M* mushroom, *H* humus, *S* soil, *W* wood debris, *O* other, *G* grassland, *P Pinus* forest (Mixed forests of *Pinus oaxacana*, *P. lawsonii*, *P. michoacana*, *P. devoniana* and *P. pringlei*), *Q Quercus* forest (Mixed forests of *Quercus magnoliifolia*, *Q. castanea*, *Q. urbanii*, *Q. rugosa*, *Q. laurina* and *Q. acutifolia*), *P-Q* forest of *Pinus* spp.-*Quercus* spp., *TDF* tropical deciduous forest, *C* crop, TG trophic group, *EM* ectomycorrhizal, *MY* mycoparasite, *S* saprobic. Mixtec community: 1: Santa Catarina Estetla; 2: San Juan Yuta; 3: San Miguel Tulancingo; 4: San Andrés Yutatio (numbers correspond to map in Fig. 1)

According to the interviewed inhabitants, there are several ways to prepare edible mushrooms. They require preparation before consumption, and usually all parts of the fruiting body are used. First, they are washed with water to remove dirt, putrefaction debris or adhered organic material. This allows the washer to verify that that all collected mushrooms are edible and thus avoid poisoning, especially for smaller species such as *Cantharellus cibarius s. l.* (***xi´i veya***), *Marasmius oreades* (***xi´i daa***) and *Agaricus campestris* (***xi´i nuu ite***). Subsequently, in the case of the complex *A. sect. caesarea* (***xi´i naa***), once roasted on the grill, people usually wash the mushrooms again to remove a yellow substance that can cause vomiting when consumed in excess; in *Boletus edulis s. l. (****xi´i taka****)*, before cooking, the pileipellis is removed because, according to the inhabitants, it has a slightly bitter taste.

**Fig. 5:**
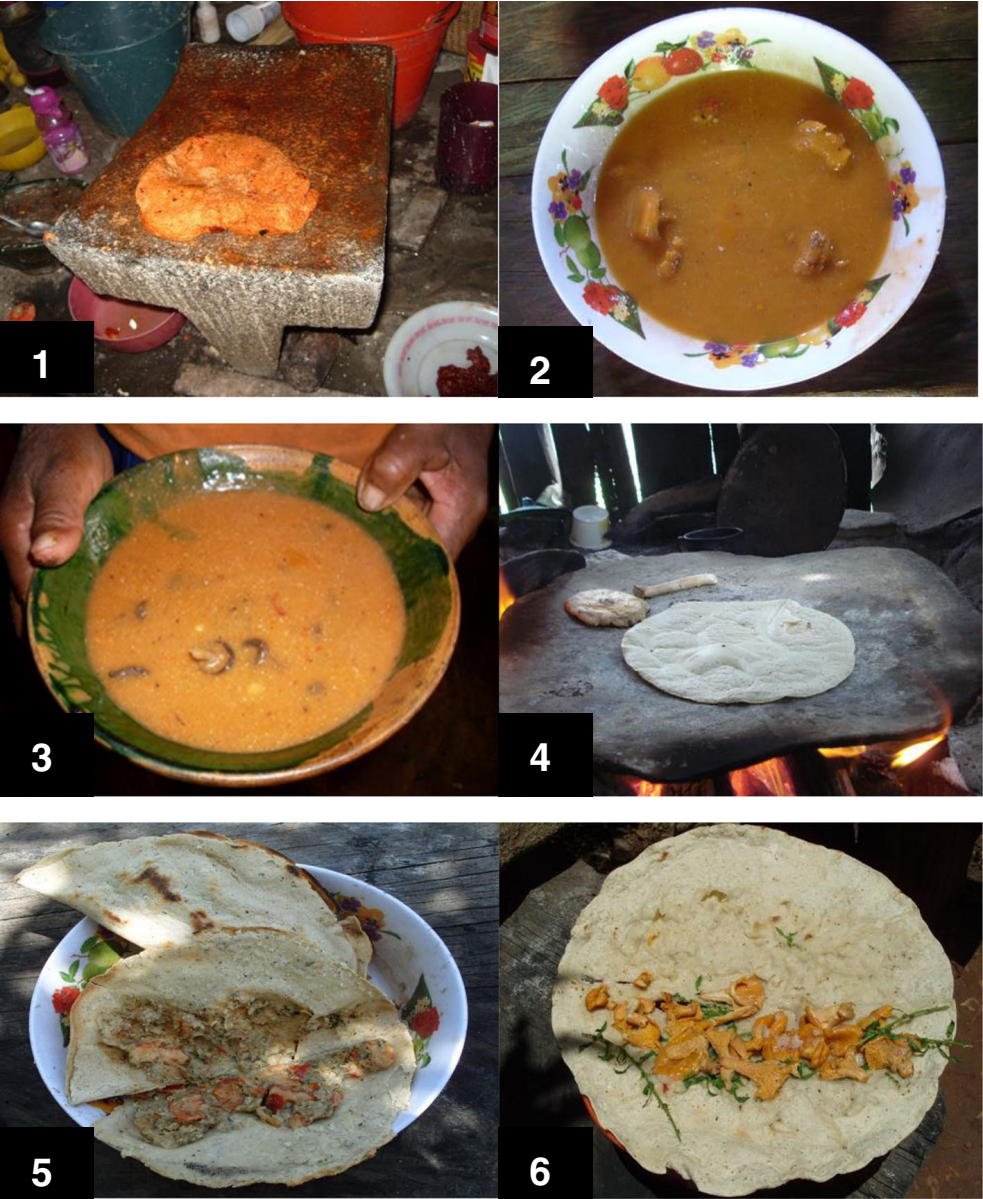
Mixtec dishes whith mushrooms: **1**. Dough for mole "amarillito"; **2**. mole "amarillito" with *Cantharellus cibarius s. l.*; **3**. mole "amarillito" with *Marasmius oreades*; **4**. *A*. aff. *jacksonii* roasted on the “grill”; **5**. Empanada with *Hypomyces lactifluorum*; **6**. Quesadilla of *Cantharellus cibarius s. l*

The method of preparing the mushrooms is related to the collected amount. When it is a small amount, they can be roasted on the griddle (***chi´o nuu xiyo***) and prepared in quesadillas (***dita kotna´tnu***, folded tortillas), empanadas (***dita xɨtɨ***, tortillas with a pouch). According to the species of mushroom, they can also be seasoned with epazote (***minu chɨ´ɨn***) (*Dysphania ambrosioides* (L.) Mosyakin et Clemants), hoja santa (***ndua ndoo***) (*Piper auritum* Kunth) or spearmint (***minu stila***) (*Mentha spicata* L*.)*. Alternatively, when enough mushrooms are available (depending on the number of family members), a more elaborated stew such as soup (***caldu xi´i***) or "amarillito" mole (***ndeyu xi´i***) is prepared. The "amarillito" mole is a stew typical of the state of Oaxaca that is prepared with ground yellow corn and flavoured with chili pepper (*Capsicum annum* L.), cloves (*Syzygium aromaticum* L.) and “hoja santa” (*P. auritum* Kunth). Species that are often prepared in "amarillito" mole are *A. campestris* (***xi´i nuu ite***), *A. pampeanus* (***xi´i nde´i***), *C. cibarius* s. l. (***xi´i veya***), *N. lepideus* (***xi´i ntaka’an ñu´u***), *P. radicata* (***xi´i tuchi***) and *M. oreades* (***xi´i daa***) (Fig. 5). Although some species can be prepared in the same way, different mushrooms are never mixed in the same dish because each differs in cooking time and flavour.

The order of preference based on taste was, according to the people interviewed the species *C. cibarius* s. l. (***xi´i veya***), *A.* sect. *caesarea* (***xi´i naa***), *N. lepideus* (***xi´i ntaka’an ñu´u***), *P. radicata* (***xi´i tuchi***) and *M. oreades* (***xi´i daa***). Mushrooms are consumed during the rainy season, without a well-defined amount. Mushroom collection is not an activity for which time is explicitly set aside but is done as people perform their everyday activities such as herding cattle, collecting firewood, hunting and traveling to cultivation parcels or neighbouring villages. Lignicolous species such as *N. lepideus* (***xi´i ntaka’an ñu´u***), *P. radicata* (***xi´i tuchi***) and *Schizophyllum commune* (***xi´i tnu kutu***)*,* once dehydrated, can be stored without losing their flavour, to be consumed in the months when no mushrooms develop. *P. radicata* is a saprophytic species colonising dead guachépil tree (*Diphysa robinioides* Benth.) roots that has traditionally been highly valued as food by the community members, so it is even dehydrated and exported in small quantities to Mixtec communities living in the United States of North America who originated in the region.

*Neolentinus lepideus* [***xi´i ntaka’a ñu´u***, thunder mushroom (when God or land returns to talk)] is considered by the Mixtec group as a "bioclimatic indicator". It is believed that it only emerges when there is a thunder and announces the rainy season. If the fungus develops in the months of February and March, that indicates that the rainy season will begin early and continue for a long period. Conversely, if it appears in the months of April or May, the rains will be delayed and the season will be short. In observations made in this study during the years 2009 to 2014, the accuracy of this belief was recorded. For the Mixtec peoples, rain (***savi*** or ***dau***) is the predominant meteorological phenomenon, and other phenomena are linked to it.

Some species have names in the region because they: i) were consumed in the past, ii) are similar to another species that is currently used; or iii) are associated with an important familiar entity in the known universe of the interviewees. For example, *Lactarius indigo* (***xi´i kuilu***) is associated with the plumage colour of the “chara” bird (*Aphelocoma woodhouseii*) (***t + daa kuilu***), which produces an alarming chant that the Mixtec peoples consider to mean that an attack is coming or something bad will happen.

#### Ludic use

In the community of Santa Catarina Estetla, the youngest children collect species of *Lycoperdon* in their immature or mature state because of their form of "small balls", and they also like to squeeze them because of the "dust" (consisting of spores) they emit. Similarly, they collect sporocarps of *Astraeus hygrometricus* (***xi’i chɨndɨɨ***, mushroom elf), which, because of their star shape, are collectibles (there is a challenge to find the most complete sporocarps and ones having different sizes). An advantage of this mushroom for ludic use is its durability. Also, informants reported that species of the genus *Calvatia* (***xi´i ndɨvɨ kuni***, mushroom egg turkey hen) or *Pisolithus* (***xi´i ndɨvɨ burru***, mushroom donkey testicle), when ripe, are used in children's games as projectiles ("snowballs") to throw at friends, siblings or grazing livestock. Undoubtedly, the natural curiosity of children represents potential and hope for the preservation and care of invaluable mycological resources for their ludic importance.

#### Hallucinogenic mushrooms

The use of hallucinogenic mushrooms in the communities under study was not reported. However, the informants mentioned that in the San Antonio Huitepec Municipality south of the Santa Catarina Estetla and San Juan Yuta communities, healers and shamans use mushrooms for divination or healing purposes.

#### Toxic mushrooms

Knowledge of toxic mushrooms has an ancestral origin in the studied communities, and it is based on local mycetismus derived from the probable consumption of species such as *A. bisporigera*, *A. verna* and *A. virosa*, which are abundant in the region and are recognised locally as deadly mushrooms. Inhabitants of Santa Catarina Estetla and San Juan Yuta classify deadly or poisonous mushrooms into three groups: i) “frog mushrooms” (***xi’i la’va***) that include *Amanita* species with a smooth, slimy and wet pileus such as *A. verna, A. phalloides* and *A. virosa*; ii) “toad mushrooms” (***xi’i la’va ndɨ’yɨ***) that include species with remains of volva or flakes (“pimples”) in the pileus, such as *Amanita muscaria* and *Lepiota* spp.; and iii) “bad or crazy mushrooms” (***xi´i kue´e***) or “not edible mushrooms”, which comprise all species not included within the two former groups that have either no known use or no known name. The term “toad mushrooms” is used to scare people, especially children, to prevent intoxication. People in the communities hold the belief that when someone approaches a toad, the animal expels urine at the observer’s eyes and that this can cause blindness.

#### Mushrooms with pharmacological potential

Within the universe of fungi present in the communities under study, some identified species have been reported to contain compounds with pharmacological and nutraceutical potential (Table 4).

**Table 4:** Species of wild mushrooms with potential use as food or medicine identified in the Mixtec studied communities

Taxa	Mixtec name	Use	Substrate	Habitat	TG	Mixtec community
Ascomycetes						
*Helvella acetabulum* (L.) Quél.	*xi’i kue´e*	E	S	Q	EM	1
*Helvella crispa* (Scop.) Fries	*xi’i kue´e*	E, PP	S	Q	EM	1
*Helvella elastica* Bull.	*xi’i kue´e*	E	S	Q	EM	1
*Helvella lactea* Boud.	*xi’i kue´e*	E	S	Q	EM	1
*Helvella lacunosa s. l.* Afzelius ex Fries.	*xi’i kue´e*	E, PP	S	P-Q, Q	EM	1
*Hypomyces macrosporus* Seaver	*xi´i lo´o ya´a*	E	M	P	MY	1
Basidiomycetes						
*Amanita fulva* Fr.	*xi´i la´ava*	E	S	Q, P-Q	EM	1
*Amanita rubescens* (Pers.: Fr.) S.F. Gray.	*xi´i la´ava ndɨ´yɨ*	E, PP	S	Q, P-Q	EM	1
*Amanita* sect. *caesarea* (Scop.:Fr.) Pers.	*xi´i naa*	PP	S	Q, P-Q	EM	1, 2
*Amanita vaginata* (Bull.) Lam.	*xi´i la´ava*	E	S	Q, P-Q	EM	1
*Armillaria tabescens* (Scop.) Emel	*xi´i yutnu*	E	W	Q, P-Q	P	1
*Astraeus hygrometricus* (Pers.: Pers.) Morgan.	*xi’i chɨndɨɨ*	PP	S	Q, G	EM	1
*Austroboletus betula* (Schwein.) E. Horak	*xi´i taka*	E	S	P, P-Q	EM	1
*Austroboletus gracilis* (Peck) Wolfe	*xi´i taka*	E	S	Q, P-Q	EM	1
*Boletellus* aff. *ananas* (M.A. Curtis) Murrill	*xi’i kue´e*	E	S	Q	EM	1
*Boletopsis grisea* (Peck) Bondartsev & Singer	*xi´i taka*	E, PP	S	Q	EM	1
*Boletus aereus* Bull.	*xi´i taka*	E	S	Q, P-Q	EM	1
*Boletus* aff. *erythropus* Pers.	*xi´i taka*	E	S	Q, P-Q	EM	1
*Boletus bicolor* Raddi	*xi´i taka*	E	S	Q, P-Q	EM	1
*Boletus edulis s.l.* Bull. ex Fr.	*xi´i taka ya´a*	PP	S	Q, P-Q	EM	1
*Boletus pinophilus* Pilát & Dermek	*xi´i taka*	E	S	Q, P-Q	EM	1
*Butyriboletus regius* (Krombh.) Arora & J.L. Frank	*xi´i taka tikue´e*	E, PP	S	Q, P-Q	EM	1
*Cantharellus “cibarius”* sp. 1	*xi´i veya*	PP	S	Q, P, P-Q	EM	1, 2
*Cantharellus “cibarius”* sp. 2	*xi´i veya*	PP	S	Q, P, P-Q	EM	1, 2
*Cantharellus cinnabarinus* (Schwein.) Schwein.	*xi’i kue´e*	E	S	Q, P-Q	EM	1
*Chroogomphus jamaicensis* (Murrill) O.K. Mill	*xi’i kue´e*	E	S	Q	EM	1
*Clavulina rugosa* (Bull.) J. Schröt	*xi´i ndɨkɨ idu*	E	S	Q	EM	1
*Clitocybe gibba* (Pers.) P. Kumm.	*xi’i kue´e*	E	H	Q	S	1
*Coprinus comatus* (O. F. Müll.) Pers.	*xi´i nuu ka´ava ntukutu*	E	H	G	S	1, 2
*Craterellus cornucopioides* (L.) Pers.	*xi’i kue´e*	E, PP	S	Q, P-Q	EM	1
*Craterellus lutescens* (Fr.) Fr.	*xi’i kue´e*	E	S	Q, P-Q	EM	1
*Craterellus tubaeformis* (Fr.) Quél.	*xi’i kue´e*	E	S	Q, P-Q	EM	1
*Frostiella russellii* (Frost) Murrill	*xi´i taka tɨkue´e*	E	S	P, P-Q	EM	1
*Hydnum repandum* L.: Fr.	*xi´i tɨntaku*	PP	S	Q, P-Q	EM	1
*Hygrophorus russula* (Schaeff.) Kauffman	*xi’i kue´e*	E, PP	S	Q, P-Q	EM	1
*Imleria* aff. *badia* (Fr.) Vizzini	*xi´i taka*	E, PP	S	Q, P-Q	EM	1
*Laccaria amethystina* Cooke	*xi’i kue´e*	E, PP	S	Q, P-Q	EM	1, 2
*Laccaria* aff. *bicolor* (Maire) Orton	*xi’i kue´e*	E, PP	S	Q, P-Q	EM	1, 2
*Laccaria laccata s. l.* (Scop.: Fr.) Cooke.	*xi’i kue´e*	E, PP	S	Q, P-Q	EM	1
*Lactarius aff. piperatus* (L.) Pers.	*xi’i kue´e*	PP	S	Q, P-Q	EM	1
*Lactarius* aff. *vellereus* (Fr.) Fr.	*xi’i kue´e*	E, PP	S	Q, P-Q	EM	1
*Lactarius indigo* (Schwein.) Fr.	*xi´i kuilu*	E	S	Q, P-Q	EM	1
*Lactarius torminosus* (Schaeff.) Gray	*xi´i kue´e*	E	S	Q, P-Q	EM	1
*Lentinus crinitus* (L.) Fr.	*xi´i yutnu*	E	W	Q, P-Q	S	1, 2
*Lycoperdon pyriforme* Schaeff.	*xi’i kue´e*	E	H	G	S	1, 2
*Lycoperdon* aff. *spadiceum* Pers.	*xi’i kue´e*	E	H	G	S	1
*Lycoperdon perlatum* Pers.	*xi’i kue´e*	E	H	G	S	1, 2
*Lyophyllum decastes* (Fr.) Singer	*xi’i kue´e*	E, PP	S	Q, P-Q	EM	1
*Macrolepiota procera* (Scop.) Singer	*xi´i la´ava ndɨ´yɨ*	E	H	P	S	1, 2
*Ramaria botrytis* (Pers.) Ricken.	*xi´i ndɨkɨ idu*	PP	S	Q	EM	1, 2
*Ramaria flava* Quel.	*xi´i ndɨkɨ idu*	PP	S	Q	EM	1, 2
*Rhizopogon roseolus* (Corda) Th. Fr.	*xi’i kue´e*	E, PP	S	Q	EM	1
*Russula brevipes* Peck.	*xi´i ya’a*	E, PP	S	Q, P-Q	EM	1
*Russula cyanoxantha* (Sch.) Fr.	*xi´i ya’a*	E, PP	S	Q, P-Q	EM	1
*Russula delica* Fr.	*xi’i kue´e*	E, PP	S	Q, P-Q	EM	1
*Russula grata* Britzelm.	*xi´i ya’a*	E	S	Q, P-Q	EM	1, 2
*Russula rosea* Pers.	*xi´i ya’a*	E	S	Q, P-Q	EM	1,
*Sparassis crispa* (Wulfen) Fr.	*xi’i kue´e*	E	W	P, P-Q	S	1
*Strobilomyces confusus* Singer	*xi’i kue´e*	E	S	Q, P-Q	EM	1
*Suillellus luridus* (Schaeff.) Murrill	*xi´i taka*	E	S	Q, P-Q	EM	1, 2
*Suillus collinitus* (Fr.) Kuntze	*xi´i taka kuaan*	E, PP	S	P, P-Q	EM	1
*Tremellodendron* schweinitzii (Peck) G.F. Atk.	*xi’i kue´e*	E	S	Q, P-Q	EM	1
*Tricholoma equestre* (L.) P. Kumm*.*	*xi’i kue´e*	E^a^, PP	S	Q, P-Q	EM	1
*Tylopilus felleus* (Bull.) P. Karst.	*xi´i taka*	E	S	Q, P-Q	EM	1, 2
*Xerocomellus chrysenteron* (Bull.) Šutara	*xi´i taka*	E	S	Q, P-Q	EM	1

^a^Despite that in general this species is considered edible and widely used as food, it can be toxic if consumed in great ammounts [98]. The nomenclature is based on *the Index fungorum* [36] and the ectomycorrhizal status in Rinaldi et al. [96] and Comandini et al. [97]. *E* edible in other regions of Mexico, *PP* with pharmacological potential, *M* mushroom, *H* humus, *S* soil, *W* wood debris, *O* other, *G* grassland, *P Pinus* forest (Mixed forests of *Pinus oaxacana*, *P. lawsonii*, *P. michoacana*, *P. devoniana* and *P. pringlei*), *Q Quercus* forest (Mixed forests of *Quercus magnoliifolia*, *Q. castanea*, *Q. urbanii*, *Q. rugosa*, *Q. laurina* and *Q. acutifolia*), *P-Q* forest of *Pinus* spp.-*Quercus* spp., *TDF* tropical deciduous forest, *C* crop, *TG* trophic group, *EM* ectomycorrhizal, *MY* mycoparasite, *S* saprobic. Mixtec community: 1: Santa Catarina Estetla; 2: San Juan Yuta; 3: San Miguel Tulancingo; 4: San Andrés Yutatio (numbers correspond to map in Fig. 1)

### Ecology, phenology and trophic groups of wild mushrooms

The timing of the onset of the reproductive phase of mushroom species is related to the rainy period. In general, it starts in February and ends in September. *N. lepideus* presents a phenological pattern of early fruiting; it can be collected in the months from February to April, whereas *A. campestris* can be found in May and early June with a prolonged early pattern.

In the mid-season months of highest precipitation, July and August, species such as *A. sect. caesarea* and *Ustilago maydis* develop, exhibiting a short phenology. In September, prolonged late-maturing species such as *C. cibarius s. l.* can be found.

Of the 116 mushroom species hitherto recorded in the study areas, 99 (93 %) species are Basidiomycetes and 7 (7 %) are Ascomycetes. Eigthy species (75 %) grow on soil/litter substrates, whereas the remaining 25 % develop on wood or other substrates, 80 species (75 %) are ectomycorrhizal, 22 species (21 %) are saprobionts and four are parasites (4 %) (Tables 3 and 4). *H. lactifluorum* and *H. macrosporus* parasitise *R. brevipes*, providing a leathery, hard consistency and orange or coffee colouring to the fungus, respectively. *N. lepideus* and *S. commune* are saprophytic species that, once dried, can be stored without losing their flavour, to be consumed during the months in which no mushroom species develop. *P. radicata* is a species that develops in the dead roots of the guachépil tree (*Diphysa robinioides* Benth.), whose development is encouraged on cropland because its flowers and young pods are also used as food.

With respect to the vegetation where fungi grow, most occur in places with pine-oak litter (*Pinus oaxacana*, *P. lawsonii*, *P. michoacana*, *P. pseudostrobus*, *P. patula*, *P. montezumae*, *Quercus magnoliifolia*, *Q. castanea*, *Q. affinis*, *Q. urbanii*, *Q. rugosa*, *Q. laurina*, Q*. acutifolia*, *Juniperus flaccida* and *Arbutus xalapensis*). Some species are found in microhabitats where pine (*Pinus* spp.) or oaks (*Quercus* spp.) predominate. The exceptions are *H. lactifluorum*, which prefers sites with a predominance of “yellow oak” (*Quercus magnoliifolia* Née) and pointleaf manzanita (*Arctostaphylos pungens* Kunth); species such as *A. campestris*, *A. pampeanus* and *M. oreades* grow in sites with secondary vegetation or pasture; and species such as *H. petaloides*, *P.* aff. *eryngii*, *P.* aff. *dryinus* and *S. commune* develop in the tropical deciduous forest where species of Copal (*Bursera* spp.), Tepehuaje tree (*Lysiloma acapulcensis* (Kunth) Benth) and round oak (*Q. glaucoides* M. Martens & Galeotti) can be found.

#### Mycophagy by wild animals

Interviewees mentioned that some wild animals, such as squirrels (*Sciurus aureogaster*) and deer (*Odocoileus virginianus oaxacensis*), consume and disseminate *A.* sect. *caesarea*, *Boletus edulis s. l. and Russula mexicana*. This can be considered a very advanced ecological notion based on strong observational skills, linked to both fungal mycophagy and subsequent dispersion caused by mammals.

### Transmission of mycological knowledge

Despite the ongoing process of acculturation in the region under study, traditional knowledge is preserved and there is transmission of that knowledge to new generations. Knowledge is passed from parents to children in a dynamic and versatile way during the daily life activities in the field, beginning at an early age (seven or eight years or sooner) when they can go to the field. The main instruction is in the places where fungal species can be found and the identification of species that are edible, toxic and have ludic uses. At the time of collecting fungi, people cut the stipe, leaving the volva or stipe base. With this, fungal growth the next year is guaranteed. People know with certainty where each species develops and visit the same sites to collect useful wild mushrooms, constituting an initial notion of the growth of "something" under the earth that produces sporomes.

### Environmental and cultural factors

The main factors that influenced the richness, knowledge and use of edible mushrooms in the four studied communities were: i) the natural vegetation cover, for example in the communities of Santa Catarina Estetla and San Juan Yuta, which have 72 and 50 % of natural pine and oak forests, respectively, there was a greater richness and therefore, knowledge and use of wild edible fungi compared with the communities of San Miguel Tulancingo and San Andrés Yutatío in which there is no coniferous forest and the oak forest occupies only 40 % (Table 5); ii) soil degradation, San Miguel Tulancingo and San Andrés Yutatío, present high levels of soil erosion originated by high deforestation rates and therefore they showed smaller diversity and richness of wild edible fungi (Table 5); iii) inter- and intra-annual rainfall influenced the fruiting season, diversity and abundance of wild edible fungi, for example in 2013 increased rainfall was distributed during the months from May to November, which caused an extension of the phenological fruiting season and higher diversity and abundance of wild edible fungi in the studied communities. In contrast in 2011 the rainy season was restricted from June to September, which resulted in low production of wild edible fungi; iv) the degree of urbanization was inversely proportional to the knowledge and use of wild edible fungi as shown by the fact that the communities San Miguel Tulancingo and San Andrés Yutatío which are urban communities use a very low number of wild mushrooms;; and v) the average number of people in extreme poverty was directly proportional to the use and knowledge of wild edible fungi in the studied Mixtec communities (Table 5). It was found that not only a single environmental or cultural characteristic, but a combination of them including higher vegetation cover of natural pine and oak forests, lower soil erosion and higher economic marginalization showed a greater richness and knowledge of wild mushrooms in the studied communities.

**Table 5:** Demographic, cultural and environmental characteristics of the four Mixtec studied communities in southeastern Mexico and richness of fungal species locally used

Name of community	Population^a^	Native Mixtec Speakers^a,b^	Natural vegetation cover^a^	Degree of soil erosion^a^	Degree of economic marginalization^a^	Richness of fungal species recognized and used
SCE	1,156	94.8 %	PQF (72 %), RA (21 %), SV (7 %)	Medium	Very high	48
SJY	432	45.3 %	PQF (50 %), RA (29 %), SV (21 %)	Medium	Very high	24
SMT	346	8.1 %	PQF (40 %), SG (46 %), SH (2 %), RA (12 %)	Very high	Medium	2
SAY	695	50.7 %	PQF (40 %), TDF (15 %), SV (33 %), RA (11 %)	High	High	2

*SCE* Santa Catarina Estetla, *SJY* San Juan Yuta, *SMT* San Miguel Tulancingo, *SAY* San Andrés Yutatio, *PQF*: natural forests of *Pinus* spp. and *Quercus* spp., *TDF* tropical deciduous forest, *RA* rainfed agriculture, *SG* secondary grassland, *SV* secondary vegetation, *SH* shrub vegetation

^a^Information based on INEGI [19], SEDESOL [23] and CONEVAL [24]

^b^Percentage of native speakers over 5-year-old

### Commercialization and potential use of wild mushrooms

There is no information on the commercialization of wild mushrooms in the studied communities. However, it is now common for people who live in the communities centre or other surrounding communities to ask farmers to obtain specific species for purchase; this occurs, for example, in the community of Santiago Huaxolotipac located in the Municipality of San Antonio Huitepec with the species *H. repandum* (***xi´i tɨntaku***, worm mushrooms). In this municipality, until recently, agricultural, livestock or harvest products such as wild mushrooms were used as currency in the regional tianguis or "open air market". Currently, during the rainy season, species such as *A.* sect. *caesarea*, *C. cibarius s. l.* and *H. repandum* are sold in the regional market of Zaachila, in the Central Valley and in the “Mercado de Abastos” [Food Market] of the city of Oaxaca. These species are collected and kept by farmers who bring their products to market, who originate in the Mixtec communities of San Miguel Peras and Santiago Tlazoyaltepec.

## Discussion

This research shows that the Mixtec communities studied have developed a complex and precise nomenclature and classification of wild mushrooms, similarly to those documented for other Mesoamerican groups including for example the Nahua [49–51], Totonac [52] Maya [53, 54] and Zapotec [8, 10] people. The richest source of ethnobiological lexicon in the Mixtec language is the dictionary compiled in 1593 by Dominican missionaries in the region of Teposcolula, state of Oaxaca [38, 55], and this is where the word "***siye***" for fungus first appears in writing. In the ethno-linguistic variants of the communities studied, there is a classification for plants, animals and fungi very similar to that recorded in 1593 in Teposcolula [38, 55] as well as to the one from San Juan Diuxi [42] in the Mixteca Alta of Oaxaca. This corroborates the assumption that the basic groups of classification recorded by Alvarado [38] remain valid in contemporary Mixtec languages.

It is interesting to note that in the case of *U. maydis* in the Mixtec variant of San Juan Diuxi it is named ***txítî***, which can be translated as "belly or stomach". This corroborates a note by De Avila [55] that in the Mixtec classifications the species *U. maydis* is not related to mushrooms. Valadez [56] mentioned that the earliest mention of *U. maydis* or "cuitlacoche" is found in a work of the sixteenth century: "General History of the Things of New Spain” by Sahagún, indicating that it is an abnormality of corn that leads the cob to acquire a blackish colour and become something like mud. This is where we find the oldest name for the fungus, "cujtlacochi", which means something like annoying dirt growing on top (of the corn). In central Mexico, the species is known as cuitlacoche or huitlacoche. Its name comes from the Nahuatl term ***cuitlacochtli***, a term composed of ***-cuitla (tl)-***, dirt, garbage, or excrement and ***-cochtli-,*** asleep, meaning therefore sleeping dirt, apparently because the spores are covered by the bracts of the parasitised spike [49]. As in the studied communities, there are other ethnic groups of Mexico that do not consider *Ustilago maydis* a mushrooms, their names for the species include: ***kjú tha*** (to lose the cob) in Otomí of the State of Mexico [50], ***xanat kuxi*** (corn flower) in Totonac of Veracruz [52], ***jaroi*** or ***jura'*** (heart) in Tepehuano of Durango [57], ***stok 'al ixim*** (corn storm clouds) in Tsotsil of Chiapas [53] and ***ta´wa nal chaak*** (excrement of the rain god) for the Mayas in the southern of México [56].

Various names for wild mushrooms are registered in the Mixtec-Spanish dictionaries published by the Summer Institute of Linguistics in Mexico without specifying the fungal species involved [38–48]. However, some fungal species can be easily identified based on the Spanish meaning and on similarities with names that are currently used in the studied communities (Table 6). For example, the term ***ji´i váyá*** or ***ji´i vaya*** refers to an edible species of the genus *Cantharellus cibarius s. l.;****xí´í yau*** refers to various edible species of the *Pleurotus* genus including *P. eryngii* and *P. dryinus*; ***ji´i*[*yika tnu_ni’ma ma*]** refers to various edible species of *Pleurotus* or to *Hohenbuehelia petaloides*; ***xiti*** is *Ustilago maydis*, ***jihì naa*** corresponds to *Amanita sect. caesarea*; ***jihì landia*** is *Lactarius indigo*; ***jihì leyu*** refers to various edible species of *Agaricus* including *A. campestris* or *A. pampeanus*; ***jihì takà*** corresponds to edible species of *Ramaria* including *R. botrytis* or *R.* aff. *flava*; ***jihì yaha*** or ***ji´i ya´a*** refers to edible species of the genus *Russula* including *R. mexicana* or *R.* aff*. cyanoxantha*, ***jihì kóhló*** corresponds to *Neolentinus lepideus*; and ***ji´i tɨndaku*** refers to *Hydnum repandum*.

**Table 6:** Generic terms related with mushrooms cited in some Mixtec dictionaries Mixtec and geographic regions of the language variants

Term	Variants of the term Mixtec and English translation	Geographic region of linguistic variation	Citation
		NW of Oaxaca	
*siye*	mushrooms	Teposcolula	[38]
*ji´i*	*ji´i váyá* “mushroom orange”	San Miguel El Grande	[39]
*xí´î*	*xí´í nda´nda idu, xí´í yau* “mushroom of maguey”, *xiti* “huitlacoche”	San Juan Diuxi	[42]
*jihì*	*jihì naa* “mushroom (large edible yellow)”, *jihì ñáá* “mushroom bad (poisonous)”, *jihì ichà* “mushroom grass”, *jihì landia* “mushroom blue”, *jihì leyu* “champiñón”, *jihì martiu* “mushroom hammer”, *jihì sòho vílu*“mushroom cat ear”, *jihì yáa sndikì* “mushroom bull tongue”*, jihì takà*“mushroom deer horn”, *jihì yaha* “mushroom of pepper”, *jihì jahà yunu*“mushroom walking stick”, *jihì shàhan* “mushroom lard”, *jihì burru*“mushroom of donkey”, *jihì kóhló* “mushroom turkey”	Chalcatongo (*ñuù ndéyá*)	[43]
*ji’i*	*ji’i chisun* “champiñón”, *ji’i* [*yika tnu_ni’ma ma*] “mushroom of Cazahuate tree”, *ji’in* [*nuu ñu’ú ma*] “mushroom of soil”	Magdalena Peñasco	[48]
		NE of Oaxaca	
*xɨ´xɨ*	mushrooms	San Juan Coatzospan	[47]
		SW of Oaxaca	
*ji´i*	*ji´i ya´a* “mushroom spicy”, *ji´i ya´a isu* “mushroom deer”, *ji´i saa nchaa* “mushroom of little bird”, *ji´i tɨndaku* “mushroom of little worms”, *ji´i vaya* “mushroom yellow”, *ji´i xini* “mushroom skull”	Santiago Yosondua	[46]
*sehie*	mushrooms	Chayuco	[40]

### Uses of fungi

#### Edible species

The number of species consumed in the studied areas is smaller than that reported in ethnomycological studies in other temperate zones of Mexico. For example, in a Nahua community in Tlaxcala in central Mexico, Montoya et al. [51] reported 30 species of edible wild mushrooms and in a Zapotec community in the state of Oaxaca, Garibay-Orijel et al. [9] reported 96 species of edible wild mushrooms. Most of the fungal species consumed by Mixtecs grow on pine and oak forest, however some of them develop in deciduous forest, including for example *Schizophyllum commune*. Some authors have reported the importance of *S. commune* in the tropical region, indicating a geographical range of consumption and sale extending from the coast of the Gulf of Mexico to the tropical zone of Guatemala. However, the records of its consumption appear to be restricted to the tropical zones with the exception of the town of Huautla de Jiménez, Oaxaca, where the climate is rather temperate humid [58]. Some edible mushrooms among Mixtecs are associated with climatic events, particularly the thunders. In the case of the sporomes of *Neolentinus lepideus* it has been consider that only are produced at the beginning of rainy season with the first thunders. The relationship between thunders and sporocarp production has long been considered a "belief"; however, it is necessary to take into account the deep mycological knowledge that different cultures have of the ecological relationships in the environments they inhabit. Several experimental studies have shown that electrical shocks have a positive response on sporocarp formation in edible mushrooms such as matsutake (*Tricholoma matsutake*) [59] and *Laccaria laccata* [60]. Fifty-six species that are consumed elsewhere in the state of Oaxaca or in the central or northern region of Mexico were identified in the study area but they are not used in the studied communities [9, 61–63]. Some of these are *Amanita crocea, A. fulva*, *A. rubescens*, *Austroboletus betula*, *Boletus frostii, Clitocybe gibba, Helvella lacunosa s. l.*, *Helvella crispa s. l*., *Hypomyces macrosporus*, *Hygrophorus russula*, *Laccaria laccata s. l.*, *Lactarius indigo*, *Lycoperdon* spp., *Lyophyllum decastes*, *Macrolepiota procera*, *Russula brevipes, R. delica*, *R.* aff. *cyanoxantha*, *Sparassis crispa* and *Suillus collinitus* (Table 4).

#### Ludic use

Ludic uses occur in other ethnic groups in Mexico, for example: i) the Chinantec people of the state of Oaxaca use *Auricularia* as a "small bag" or "small balloon" by separating the membranes and making a small hole [64]; ii) the Lacandon peoples of Lacanjá-Chansayab in the state of Chiapas use the fungus called *Chak ach* (*Cookeina sulcipes* and *C. tricholoma*) to hear sounds by blowing into the cup and then putting it on the ear [54]; and iii) the Zapotec people of Oaxaca use *Ganoderma applanatum* for making prints of animals, plants and/or landscapes [10]. Undoubtedly, the natural curiosity of children represents potential and hope for the preservation and care of invaluable mycological resources for their ludic importance.

#### Hallucinogenic mushrooms

This could be related to rituals of pre-Hispanic heritage mentioned in the *Codex Yuta Tnoho* (Santiago Apoala), given that the locations were established in the Post classic period (950–1520 A.D.) before the arrival of the Spaniards, when the Mixtec culture reached its peak [65]. In that regard, Ravicz [66] mentioned the use of neurotropic mushrooms in the Mixteca Alta region, though the communities where this practice was followed were not identified. The limited current use of species of hallucinogenic mushrooms from the genus *Psilocybe* for sacred or divination purposes in the Mixtec group contrasts with: i) the great diversity of these species in the state of Oaxaca, which contains 27 of the 53 hallucinogenic species of *Psilocybe* known in Mexico [67], as well as ii) the use of said mushrooms in pre-Hispanic rituals, documented in the *Codex Yuta Tnoho*, which describes a sacred ceremony in which various Mixtec deities consumed sacred mushrooms prior to the first sunrise [14, 15]. One factor that may have influenced the decline in the use of sacred mushrooms could be the religious persecution to which pre-Christian practices were subjected upon the arrival of Christianity in the region [16].

#### Toxic mushrooms

Despite the fact that in general the criteria used by Mixtecs, to distinguish edible and toxic mushrooms are in general accurate, some general principles have limitations given that some edible species, such as *Amanita crocea* and *A. rubescens,* are included in the “frog” or “toad” mushrooms groups and are considered toxic in the region despite being widely consumed in other regions of Mexico [8–10, 51, 62, 63]. *A. muscaria* has the erroneous reputation in the study locations of being a very poisonous mushroom; in fact, it only produces mycetismus of the gastrointestinal type [68], causing temporary vomiting and diarrhoea. In addition, this mushroom causes neurotropic activity with perceptions of hallucinations caused by its muscarine content, a glycoside and ibotenic acid, an indolic substance [69]. Wasson [18] mentioned sporadic coincidences between toads and entheogenic mushrooms in the Basque region in Spain (“toad mushroom”), in rural France and in China (“toad mushroom”). This same association is presented in the communities being studied, where the designation “toad mushroom” or “frog mushroom” is given to any species of the genus *Amanita* and to any species to be wary of. In 1953, in a journey through Mayan lands, Wasson [18] discovered the convergence of three meanings in one Mayan word: “toad”, “mushroom” and “external female genitalia”. In the community under study, something similar occurs with the word *lava* that relates to female genitalia, the mention of which causes hilarity or disgust among people.

#### Mushrooms with pharmacological potential

Today, mushrooms are valued for their nutritional value, which is attributed to their high levels of protein, fibre, carbohydrates, vitamins and minerals and low levels of fat. Some species have also been used to increase human longevity and quality of life via their medicinal and nutraceutical properties [70]. Within the universe of fungi present in the communities under study, some species consumed by the Mixtec were identified that contain compounds with pharmacological and nutraceutical potential. Additionally, species containing bioactive antioxidant compounds such as tocopherols, ascorbic acid, neogrifolin, phenolic compounds (protocatechuic, gallic, gentisic, vanillic and tannic acids) and organic acids (oxalic, malic, citric and fumaric acids) have been reported. These include *Amanita caesarea* (Scop.) Pers. [71], *Albatrellus ovinus* (Schaeff.) Kotl. et Pouzar [72], *Boletus edulis* Bull. [73], *Cantharellus cibarius* Fr. [74], *Helvella lacunosa* Afzel [75], *Hydnum repandum* L. [76], *Laccaria laccata* (Scop.) Cooke [77], *Ramaria botrytis* (Pers.) Ricken [78] y *Ramaria flava* (Schaeff.) Quél. [79]. In other species consumed in the study areas, such as *Hydnum repandum* L. [80], *Laccaria laccata* (Scop.) Cooke [80], *Lyophyllum decastes* (Fr.) Singer [81], *Ramaria flava* (Schaeff.) Quél. [79] y *Russula cyanoxantha* (Schaeff.) Fr. [82], antitumor properties have been reported. Also, anti-inflammatory properties have been reported in *Cantharellus cibarius* Fr. [83] and *Russula cyanoxantha* (Schaeff.) Fr. [82]; antibacterial properties with *Ramaria botrytis* (Pers.) Ricken [84] and *Ramaria flava* (Schaeff.) Quél. [79] and anti-HIV properties with *Hygrophorus russula* (Schaeff.) Kauffman [85].

#### Mycophagy by wild animals

Mixtec people know that some wild animals consume and disseminate some edible mushrooms. Previously, the consumption of some species of hypogeal fungi by animals such as the agouti (*Dasyprocta mexicana*) [86] and wild mouse (*Peromyscus alstoni*, *Reithrodontomys megalotis* and *Microtus mexicanus*) [87] has been recorded in Mexico. Castillo-Guevara et al. [88] showed that species of wild mice (*Neotomodon alstoni*, *Peromyscus maniculatus* and *P. alstoni*) consume fungi such as *Laccaria trichodermophora*, *Suillus tomentosus* and *Russula cuprea* and found that spore viability is not affected by consumption, suggesting that these may be effective dispersers of spores of fungal species.

### Transmission of mycological knowledge

A preservation of tradicional knowledge among Mixtecs was recorded in the studied communities. Caballero [65] mentioned that in the community of San Antonio Huitepec in the Eastern Mixteca Alta region, from six or seven years of age, a child knows how to distinguish edible wild mushrooms without equivocation or fear. At the time of collecting fungi, people cut the stipe, leaving the volva or stipe base. With this, fungal growth the next year is guaranteed. People know with certainty where each species develops and visit the same sites to collect useful wild mushrooms, constituting an initial notion of the growth of "something" under the earth that produces sporomes.

### Commercialization and potential use of wild mushrooms

In the community under study there are species with potential to be exploited in a sustainable way as a non-timber forest product for export to international markets, which could be an alternative for the conservation of mycological knowledge and resources. International trade in wild mushrooms is valued at billions of dollars annually. One reason for this high cost is that most species cannot be cultivated [89] and are of great interest for gourmet cooking in various European countries and North America [90]. Among the species with potential are *Cantharellus cibarius s. l.* and *Boletus edulis s. l.*, which have estimated annual values according to the retail market of $1.67 billion and more than $250 million U.S. dollars, respectively. Additionally, *Amanita* sect. *caesarea* also has an international market. The main countries that demand these species are Canada, France, Italy, Spain, USA, China and Germany [91, 92].

Sustainable management of wild mushrooms among the Mixtecs would be enhanced by implementing the following strategies: i) transmission of technologies to local people in order to give an added value to the commercialization of mushrooms in the region, by methods such as dehydration, preparation of brines and vinaigrettes; ii) dissemination of existing knowledge of mushrooms growing in local communities with value in international markets, which can be an important source of economic resources locally; iii) promotion of use of species that are not consumed in the region and have edible potential; iv) development of mycotourism involving the local population, including activities such as mycological tours and mycogastronomy; v) biotechnological applications of mycological resources, including cultivation of saprophytic species of biocultural importance regionally, inoculation of edible ectomycorrhizal fungi in native tree species tending to reforestation of degraded areas and development of mycosilviculture including forest management practices tending to increase natural production of wild mushrooms, mainly ectomycorrhizal species; and vi) upgrading and strengthening of ecological, cultural and socioeconomic importance of mycological resources, through activities such as fairs, exhibitions and local culinary samples, with the participation of members of the Mixtec communities, local and national government and non-governmental organizations. Some of these strategies have been successfully developed and applied in several European and Asian regions, contributing to forest conservation and sustainable management of mycological resources and natural ecosystems [93–95].

## Conclusions

This ethnomycological study is the first to focus on the Mixtec group, which is the third-largest in Mexico after the Nahua and the Maya.

Traditional knowledge of fungi in the communities of Santa Catarina Estetla and San Juan Yuta is of high accuracy from the western taxonomic and ecological perspective. Currently there is an important preservation and oral transmission of mycological knowledge to new generations of Mixtec people particularly in these later communities. The inhabitants of these localities can distinguish and name the parts of the fungi with high precision; group and assign one or more common names to edible or poisonous mushrooms; and pinpoint exactly the habitat and phenology of the species they use.

Differencial environmental, soeconomic and cultural factors among the four studied communities affect the richness, knowledge and use of edible wild mushrooms.

Despite the strong existing acculturation processes and migration in the region, the ability to recover local, traditional ethnomycological knowledge is dynamic and survives among the Mixtec peoples of Santa Catarina Estetla and San Juan Yuta. The mechanisms through which traditional mycological knowledge is maintained and adapted include manteinance of cultural identity, forest protection, preservation native language and also paradoxically through the current socieconomical marginality among the Mixtec people. In this framework, the use and sustainable management of wild mushrooms could be an alternative for local integrated development, but only if the Mixtec worldview is incorporated into regional programs.

## References

[1] Llorente-Bousquets J, Ocegueda S, Soberanes J, Halfter G, Llorente-Bousquets J (2008). Estado del conocimiento de la biota. Capital natural de México: Conocimiento actual de la biodiversidad.

[2] Hawksworth DL (2001). The magnitude of fungal diversity: the 1.5 million species estimate revisited. Microbiol Res.

[3] Guzmán G (2008). Análisis de los estudios sobre los Macromycetes de México. Revista Mexicana de Micología.

[4] INALI-DOF (2010). Programa de Revitalización, Fortalecimiento y Desarrollo de las Lenguas Indígenas Nacionales 2008–2012, PINALI.

[5] Ruan-Soto F, Garibay-Orijel R, Cifuentes J (2006). Process and dinamycs of traditional selling wild edible mushrooms in tropical México. J Ethnobiol Ethnomed.

[6] Guzmán G (2008). Diversity and use of traditional Mexican medicinal fungi. A review. Int J Med Mushrooms.

[7] BA. La clasificación de la vida en las lenguas de Oaxaca. In: García-Mendoza AJ, Ordóñez MJ, Briones-Salas MA, editors. Biodiversidad de Oaxaca. México D.F.: Instituto de Biología, UNAM-Fondo Oaxaqueño para la Conservación de la Naturaleza-WWF; 2004. p. 481–539.

[8] Garibay-Orijel R, Cordova J, Cifuentes J, Valenzuela R, Estrada TA, Kong A (2009). Integrating wild mushrooms use into a model of sustainable management for indigenous community forests. For Ecol Manage.

[9] Garibay-Orijel R, Martínez-Ramos M, Cifuentes J (2009). Disponibilidad de esporomas de hongos comestibles en los bosques de pino-encino de Ixtlán de Juárez, Oaxaca. Revista Mexicana de Biodiversidad.

[10] Garibay-Orijel R (2009). Los nombres zapotecos de los hongos. Revista Mexicana de Micología.

[11] Katz E, Rojas T (1990). Prácticas agrícolas en la Mixteca Alta. Agricultura indígena: pasado y presente.

[12] Katz E, Vargas LA (1990). Cambio y continuidad en la alimentación de los mixtecos. Anales de Antropología.

[13] Mindek D (2003). Mixtecos. Pueblos Indígenas del México Contemporáneo.

[14] Jansen MERGN, Pérez JGA (2004). Renaming the Mexican Codices. Ancient Mesoamerica.

[15] Furst JL (1978). Codex Vindobonensis Mexicanus I: A commentary.

[16] SepúlvedayHerrera MT (1999). Procesos por idolatría al cacique, gobernadores y sacerdotes de Yanhuitlán, 1544–1546.

[17] Smith ME (1973). Picture writing from Sothern Mexico; Mixtec place signs and maps.

[18] Wasson RG (1983). El hongo maravilloso: Teonanacatl. Micolatría en Mesoamérica.

[19] INEGI (National Institute of Statistics, Geography and Informatics) (2010). Compendio de información geográfica municipal de los Estados Unidos Mexicanos.

[20] Rzedowski J (1978). Vegetación de México.

[21] Sandoval CC (2002). Investigación cualitativa. Programa de especialización teórica, métodos y técnicas de investigación social.

[22] Bernard HR (2006). Research methods in anthropology: qualitative and quantitative approaches.

[23] SEDESOL (Ministry of Social Development). Cédulas de Información Municipal para el programa para el Desarrollo de Zonas Prioritarias (PDZP) en México. [http://www.microrregiones.gob.mx/]. Accessed 1 Sept 2016.

[24] CONEVAL (National Council for the Evaluation of Social Development Policies). índice de rezago social 2015 a nivel nacional, estatal y municipal en México. [http://coneval.org.mx/Paginas/principal.aspx]. Accessed 1 Sept 2016.

[25] Cifuentes J, Villegas M, Pérez-Ramírez L, Lot A, Chang F (1986). Hongos. Manual de herbario.

[26] Largent DC, Johnson D, Watling R (1980). How to Identify Mushrooms to genus III: Microscopic Features.

[27] Tulloss RE (1994). Type studies in *Amanita* section *Vaginatae* I: Some taxa described in this Century (studies 1–23) with notes on description of spores and refractive hyphae in *Amanita*. Mycotaxon.

[28] Lincoff GH (1981). The Audobon Society Fiel Guide to North American Mushrooms.

[29] Pérez-Silva E, Herrera T (1991). Iconografía de macromicetos de México. I Amanita.

[30] Singer R, García J, Gómez LD (1990). The Boletineae of Mexico and Central America. I-II. Nova Hedwig Beih.

[31] Singer R, García J, Gómez LD (1991). The Boletineae of Mexico and Central America III. Nova Hedwig Beih.

[32] Singer R, García J, Gómez LD (1992). The Boletineae of Mexico and Central America IV. Nova Hedwig Beih.

[33] Guzmán G, Ramírez-Guillén F (2001). The Amanita caesarea-complex.

[34] Kirk PM, Cannon PF, David JC, Stalpers JA (2001). Ainsworth & Bisby´s Diccionary of the Fungi.

[35] García-Jiménez J, Singer R, Estrada E, Garza-Ocañas F, Valenzuela R (2013). Dos especies nuevas del género *Boletus* (Boletales: Agaricomycetes) en México. Revista Mexicana de Biodiversidad.

[36] Index Fungorum. [http://www.indexfungorum.org]. Accessed 1 Sept 2016.

[37] USDA, ARS. National Genetic Resources Program: Germplasm Resources Information Network-(GRIN). [http://www.ars-grin.gov/]. Accessed 1 Sept 2016.

[38] De Alvarado F (1962). 1593. Vocabulario en Lengua Mixteca.

[39] Dyk A, Stoudt B (1973). Vocabulario mixteco de San Miguel El Grande.

[40] Pensinger BJ (1974). Diccionario mixteco del pueblo de Chayuco (Este de Jamiltepec).

[41] SEP (1979). Abecedario de Mixteco de Peñoles.

[42] Kuiper HA (2003). Ita, ku´u, yau, yua, yuku, yutnu, xi´i; diccionario enciclopédico de plantas. Mixteco de San Juan Diuxi y Santiago Tilantongo.

[43] Pérez JGA (2003). Sahìn Sàu. Curso de Lengua Mixteca (variante de Ñuù Ndéyá).

[44] Ferguson J (2007). Gramática popular del mixteco del municipio de Tezoatlán, San Andrés Yutatío, Oaxaca.

[45] Caballero MG (2011). Diccionario del idioma mixteco. Tutu Tu´un Ñuu Savi.

[46] Beaty FK, García SP, García SR, Ojeda SJ, San Pablo GA, Santiago JA (2012). Diccionario Básico del Mixteco de Yosondua.

[47] Small WP, Turner GJ. *Nakuā´a o tú´un kō*: Leamos nuestro idioma. Mixteco de San Juan Coatzospan. 2012.

[48] Erickson E (2013). Gramática del mixteco de Magdalena Peñasco (Sa´an ñuu savi).

[49] Del Campo MR (1968). Contribución al conocimiento de la nomenclatura micológica Náhuatl. Bol lnf Soc Mex Mic.

[50] Estrada-Torres A, Aroche RM (1987). Acervo etnomicológico en tres localidades del Municipio de Acambay, Estado de México. Revista Mexicana de Micología.

[51] Montoya A, Hernández N, Mapes C, Kong A, Estrada-Torres A (2008). The collection and sale of wild mushrooms in a community of Tlaxcala, México. Econ Bot.

[52] Chacón S (1988). Conocimiento etnoecológico de los hongos en Plan de Palmar, Municipio de Papantla, Veracruz. México Micología Neotropical Aplicada.

[53] Shepard GH, Arora D, Lampman A (2008). The grace of the flood: classification and use of wild mushrooms among the highland maya of Chiapas. Econ Bot.

[54] Ruan-Soto F, Cifuentes J, Mariaca R, Limón F, Pérez-Ramírez L, Sierra-Galván S (2009). Uso y manejo de hongos silvestres en dos comunidades de la Selva Lacandona, Chiapas, México. Revista Mexicana de Micología.

[55] BA DÁ, Sarukhán KJ, Soberon J, Halffter G, Llorente-Bousquets J (2008). La diversidad lingüística y el conocimiento etnobiológico. Capital natural de México: Conocimiento actual de la biodiversidad.

[56] Valadez R, Moreno-Fuentes A, Gómez G (2011). Cujtlacochi, El Huitlacoche.

[57] González-Elizondo M (1991). Ethnobotany of the southern Tepehuan of Durango, México: l. Edible Mushrooms. J Ethnobiol.

[58] Olivo-Aranda F, Herrera T (1994). Las especies de *Schizophyllum* en México, su distribución ecológica y su importancia etnomicológica. Revista Mexicana de Micología.

[59] Islam F, Ohga S (2012). The response of fruit body formation on *Tricholoma matsutake* In Situ condition by applying electric pulse stimulator. ISRN Agronomy.

[60] Ohga S, Iida S (2001). Effect of electric impulse on sporocarp formation of ectomycorrhizal fungus *Laccaria laccata* in Japanese red pine plantation. J For Res.

[61] Pérez-Silva E, Esqueda M, Herrera T, Coronado M (2006). Nuevos registros de Agaricales de Sonora, México. Revista Mexicana de Biodiversidad.

[62] Pérez-Moreno J, Martínez-Reyes M, Yescas-Pérez A, Delgado-Alvarado A, Xoconostle-Cázares B (2008). Wild mushrooms markets in Central México and a case study at Ozumba. Econ Bot.

[63] Estrada-Martínez E, Guzmán G, Cibrián TD, Ortega PR (2009). Contribución al conocimiento etnomicológico de los hongos comestibles silvestres de mercados regionales y comunidades de la Sierra Nevada (México). Rev Interciencia.

[64] Ruan-Soto F, Garibay-Orijel R, Cifuentes J (2004). Conocimiento Micológico Tradicional en la Planicie Costera del Golfo de México. Revista Mexicana de Micología.

[65] Caballero JJ (2009). *Ñuu davi yuku yata*. Comunidad, identidad y educación en la Mixteca (México).

[66] Ravicz R (1960). La Mixteca en el estudio comparativo del hongo alucinante. Anales del INAH.

[67] Ramírez-Cruz V, Guzmán G, Ramírez-Guillen F (2006). Las especies del género *Psilocybe* conocidas del estado de Oaxaca, su distribución y relaciones étnicas. Revista Mexicana de Micología.

[68] Guzmán G (1980). Las intoxicaciones producidas por hongos. Ciencia y Desarrollo (CONACYT).

[69] Schultes RE, Hofmann A (1982). Plantas de los Dioses. Orígenes del uso de los alucinógenos.

[70] Chang ST, Miles PG (2004). Mushrooms: Cultivation, nutritional value, medicinal effect, and environmental impact.

[71] Sarikurkcu C, Tepe B, Semiz DK, Solak MH (2010). Evaluation of metal concentration and antioxidant activity of three edible mushrooms from Mugla, Turkey. Food Chem Toxicol.

[72] Nukata M, Hashimoto T, Yamamoto I, Nobukilwasaki, Tanaka M, Asakawa Y (2002). Neogrifolin derivatives possessing anti-oxidative activity from the mushroom *Albatrellus ovinus*. Phytochemistry.

[73] Li N, Ng TB, Wong JH, Qiao JX, Zhang YN, Zhou R, Chen RR, Liu F (2013). Separation and purification of the antioxidant compounds, caffeic acid phenethyl ester and caffeic acid from mushrooms by molecularly imprinted polymer. Food Chem.

[74] Akata I, Ergönül B, Kalyoncu F (2012). Chemical compositions and antioxidant activities of 16 wild edible mushroom species grown in Anatolia. Int J Pharmacol.

[75] Leal RA, Barros L, Barreira JCM, Sousa MJ, Martins A, Santos-Buelga C, Ferreira ICFR (2013). Portuguese wild mushrooms at the “pharma-nutrition” interface: Nutritional characterization and antioxidant properties. Food Res Int.

[76] Puttaraju NG, Venkateshaiah SU, Dharmesh SM, Urs SM, Somasundaram R (2006). Antioxidant activity of indigenous edible mushrooms. J Agric Food Chem.

[77] Egwin EC, Elem RC, Egwuche RU (2011). Proximate composition, phytochemical screening and antioxidant activity of ten selected wild edible Nigerian mushrooms. Am J Food Nutr.

[78] Barros L, Dueñas M, Ferreira ICFR, Baptista P, Santos-Buelga C (2009). Phenolic acids determination by HPLC-DAD-ESI/MS in sixteen different Portuguese wild mushrooms species. Food Chem Toxicol.

[79] Liu K, Wang J, Zhao L, Wang Q (2013). Anticancer, antioxidant and antibiotic activities of mushroom *Ramaria flava*. Food Chem Toxicol.

[80] Chung KS, Eung CHCH, Byong KK, Yang SK, Yong HP (1982). Studies on the constituents and culture of Korean basidiomicetes. Arch Pharm Res.

[81] Ukawa Y, Ito H, Hisamatsu M (2000). Antitumor effects of (1 → 3)- β -D-Glucan and (1 → 6)-β-D-Glucan purified from newly cultivated mushroom, Hatake shimeji (*Lyophyllum decastes* Sing.). J Biosci Bioeng.

[82] Zhao YY, Shen X, Chao X, Ho CC, Cheng XL, Zhang Y, Lin RC, Du KJ, Luo WJ, CHEN JY, Sun WJ (2011). Ergosta-4,6,8(14),22-tetraen-3-one induces G2/M cell cycle arrest and apoptosis human hepatocellular carcinoma HepG2 cells. Biochim Biophys Acta.

[83] Moro C, Palacios I, Lozano M, D’arrigo M, Guillamón E, Villares A, Martínez JA, García-Lafuente A (2012). Anti-inflammatory activity of methanolic extracts from edible mushrooms in LPS activated RAW 264.7 macrophages. Food Chem.

[84] Alves MJ, Ferreira ICFR, Martins A, Pintado M (2012). Antimicrobial activity of wild mushroom extracts against clinical isolates resistant to different antibiotics. J Appl Microbiol.

[85] Zhu M, Xu L, Chen X, Ma Z, Wang H, Ng TB (2013). A novel ribonuclease with HIV-1 reverse transcriptase inhibitory activity from the edible mushroom *Hygrophorus rusula*. Appl Biochem Biotechnol.

[86] Estrada-Croker C, Naranjo EJ, Miller B (1996). The Mexican agouti in Chiapas, México. Inter Zool News.

[87] Valenzuela VH, Herrera T, Gaso MI, Pérez–Silva E, Quintero E (2004). Acumulación de radiactividad en hongos y su relación con los roedores en el bosque del centro nuclear de México. Revista Internacional de Contaminación Ambiental.

[88] Castillo-Guevara C, Lara C, Pérez G (2012). Micofagia por roedores en un bosque templado del centro de México. Revista Mexicana de Biodiversidad.

[89] Yun W, Hall IR (2004). Edible ectomicorrhizal mushrooms: challenges and achievements. Can J Bot.

[90] Karwa A, Vamma A, Rai M, Rai M, Varma A (2011). Edible Ectomycorrhizal Fungi: Cultivation, Conservation and Challenges. Soil Biology: Diversity and Biotechnology of Ectomycorrhizae.

[91] Hall IR, Wang Y, Amicucci A (2003). Cultivation of edible ectomycorrhizal mushrooms. Trends Biotechnol.

[92] Arora D, Dunham SM (2008). A new, commercially valuable Chanterelle species, *Cantharellus californicus* sp. nov., associated with live oak in California, USA. Econ Bot.

[93] Savoie JM, Largeteau ML (2011). Production of edible mushrooms in forests: trends in development of a mycosilviculture. Appl Microbiol Biotechnol.

[94] Martínez de Aragón J, Riera P, Giergiczny M, Colinas C (2011). Value of wild mushroom picking as an environmental service. For Policy Manag.

[95] Martínez-Peña F, de-Miguel S, Pukkala T, Bonet JA, Ortega-Martínez P, Aldea J, Martínez de Aragón J (2012). Yield models for ectomycorrhizal mushrooms in Pinus sylvestris forests with special focus on *Boletus edulis* and *Lactarius* group *deliciosus*. For Ecol Manage.

[96] Rinaldi AC, Comandini O, Kuyper TW (2008). Ectomycorrhizal fungal diversity: separating the wheat from the chaff. Fungal Divers.

[97] Comandini O, Rinaldi AC, Uyper TW, Pagano M (2012). Measuring and estimating ectomycorrhizal fungal diversity: a continuous challenge. Mycorrhiza: occurrence in natural and restored environments.

[98] Nieminen P, Kärjä V, Mustonen AM (2008). Indications of hepatic and cardiac toxicity caused by subchronic *Tricholoma flavovirens* consumption. Food Chem Toxicol.

